# Emodin exhibits anti-acne potential by inhibiting cell growth, lipogenesis, and inflammation in human SZ95 sebocytes

**DOI:** 10.1038/s41598-023-48709-x

**Published:** 2023-12-07

**Authors:** Si Liu, Xiao-Hua Luo, Yu-Feng Liu, Christos C. Zouboulis, Ge Shi

**Affiliations:** 1 Department of Cosmetic and Plastic Surgery, The Sixth Affiliated Hospital, Sun Yat-Sen University, Guangzhou, China; 2 Biomedical Innovation Center, The Sixth Affiliated Hospital, Sun Yat-Sen University, Guangzhou, China; 3 Departments of Dermatology, Venereology, Allergology and Immunology, Staedtisches Klinikum Dessau, Brandenburg Medical School Theodor Fontane and Faculty of Health Sciences Brandenburg, Dessau, Germany

**Keywords:** Cell biology, Molecular medicine

## Abstract

Emodin, a natural anthraquinone derivative, possesses anti-proliferative and anti-inflammatory properties in skin diseases. However, little information is available on the efficacy of emodin in treating acne vulgaris (acne). This study aims to investigate the protective effects and potential mechanisms of emodin as an anti-acne agent. In vitro, SZ95 sebocytes was chose to establish an acneigenic cellular model. We found that emodin effectively inhibited proliferation, induced cell cycle arrest and apoptosis of SZ95 sebocytes in a dose-dependent manner. To evaluate the lipid-lowering potential of emodin, we examined the levels of lipid contents and lipogenic transcription factors, and found that both lipid production and protein expression of PPARγ, LXR α/β, and SREBP-1 were decreased after treatment with emodin. Furthermore, our results revealed that emodin inhibited sebaceous lipogenesis induced by insulin-like growth factor 1 (IGF-1), which was accompanied by a potent inhibition of the phosphoinositide-3-kinase (PI3K)/Akt/forkhead box protein O1 (FoxO1) pathway. In detail, emodin augmented the inhibitory effect of isotretinoin and PI3K inhibitor LY294002, while attenuating the activation of IGF-1 on PI3K/Akt/FoxO1 pathway. In addition, emodin could decrease the secretion of pro-inflammatory cytokines IL-6 and IL-8, and suppress the expression of NLRP3, capase-1, IL-1β, and IL-18 in SZ95 sebocytes exposed to *Cutibacterium acnes*. Overall, our study provides preliminary evidence supporting the anti-growth, anti-lipogenic and anti-inflammatory properties of emodin, indicating the potential therapeutic application of emodin for acne treatment.

## Introduction

Acne vulgaris (acne) remains the most prevalent chronic skin disorder worldwide, affecting an estimated 9.4% of the global population. Different clinical presentations of acne include comedones, papules, pustules, and cystic nodules that may lead to scarring or hyperpigmentation^1^. The onset and development of this disease are contingent upon the pilosebaceous units. Basically, the acne pathogenesis involves four major events: hyperseborrhea, follicular hyperkeratosis, invasion of virulent *Cutibacterium acnes* (*C. acnes*), and perifollicular inflammation^2^. Acne lesions occur more frequently in areas with a higher density of sebaceous glands (SGs). Numerous studies ascertained a positive correlation between excessive sebum secretion and acne severity^3^.

SG is a multilobular holocrine-secreting appendage of the epidermis. Sebocytes are primarily responsible for synthesizing neutral lipids known as sebum. Terminal-differentiated sebocytes lyse and secret lipids through the hair follicle apertures onto the skin surface^4^. Growth hormone (GH) and lipogenic transcription factors, such as peroxisome proliferator-activated receptor (PPAR) γ, liver X receptor (LXR) α/β and sterol response element binding protein (SREBP)-1, primarily contribute to the differentiation of sebocyte^5^. Western diets, characterized by glycemic overload and milk consumption, stimulate the hepatic production of insulin-like growth factor 1 (IGF-1). IGF-1 is considered as a key trigger to the development of acne, primarily due to its ability to enhance the functioning of sebaceous glands (SGs), resulting in heightened sebocyte proliferation, differentiation, and lipid synthesis^6^. Moreover, the cutaneous commensal *C. acnes* is also a major pathophysiological factor for the follicular inflammation in the pathogenesis of acne. The pilosebaceous follicles provide anaerobic growth conditions for this microorganism and, at the same time, it can degrade abundant sebum lipids as a nutritional source^7^. These specific metabolic features render them inclined to colonize in lipid-rich sebaceous sites. In acne lesions, *C. acnes* invasion stimulates sebaceous gland activity, resulting in excessive sebum secretion and the release of IL-6, IL-1β, IL-8, TNF-α^8^. It has been estimated that *C. acnes* enhances sebocytes to produce inflammatory cytokines through the pattern recognition receptors such as Nod-like receptors (NLRs)^9^.

Of all the systematic anti-acne agents, isotretinoin (13-*cis* retinoic acid, 13-*cis* RA) is the most effective suppressor of sebum secretion, while exhibiting a risk of teratogenicity^10^. Emodin (1,3,8-trihydroxy-6-methyl-anthraquinone), a plant-derived anthraquinone derivative in the *Polygonaceae* family, carries a broad spectrum of bioactivities against tumors, obesity, inflammation and allergies^11^. While in vitro, emodin exerts an inhibitory effect on cell growth and survival by suppressing proliferation, inducing cell cycle arrest and apoptosis in various cell types^12^. In adipose tissue, emodin reduces lipid accumulation by lowering insulin levels and inhibiting the SREBP pathway^13,14^. Also, this compound has therapeutic efficacy in many cutaneous inflammatory disorders such as psoriasis and atopic dermatitis^15,16^, while its pharmacological effects on regulating lipogenesis and inflammation in sebaceous cells remain largely unknown.

The present study aimed to elucidate the possible mechanisms by which emodin exerted anti-acne potential in vitro. Specifically, we investigated the effects of emodin on proliferation, cell cycle, apoptosis, IGF-1-induced lipogenesis and *C. acnes-*induced inflammation in human SZ95 sebocytes.

## Results

### Emodin suppresses the proliferation of human SZ95 sebocytes

**Figure 1:**
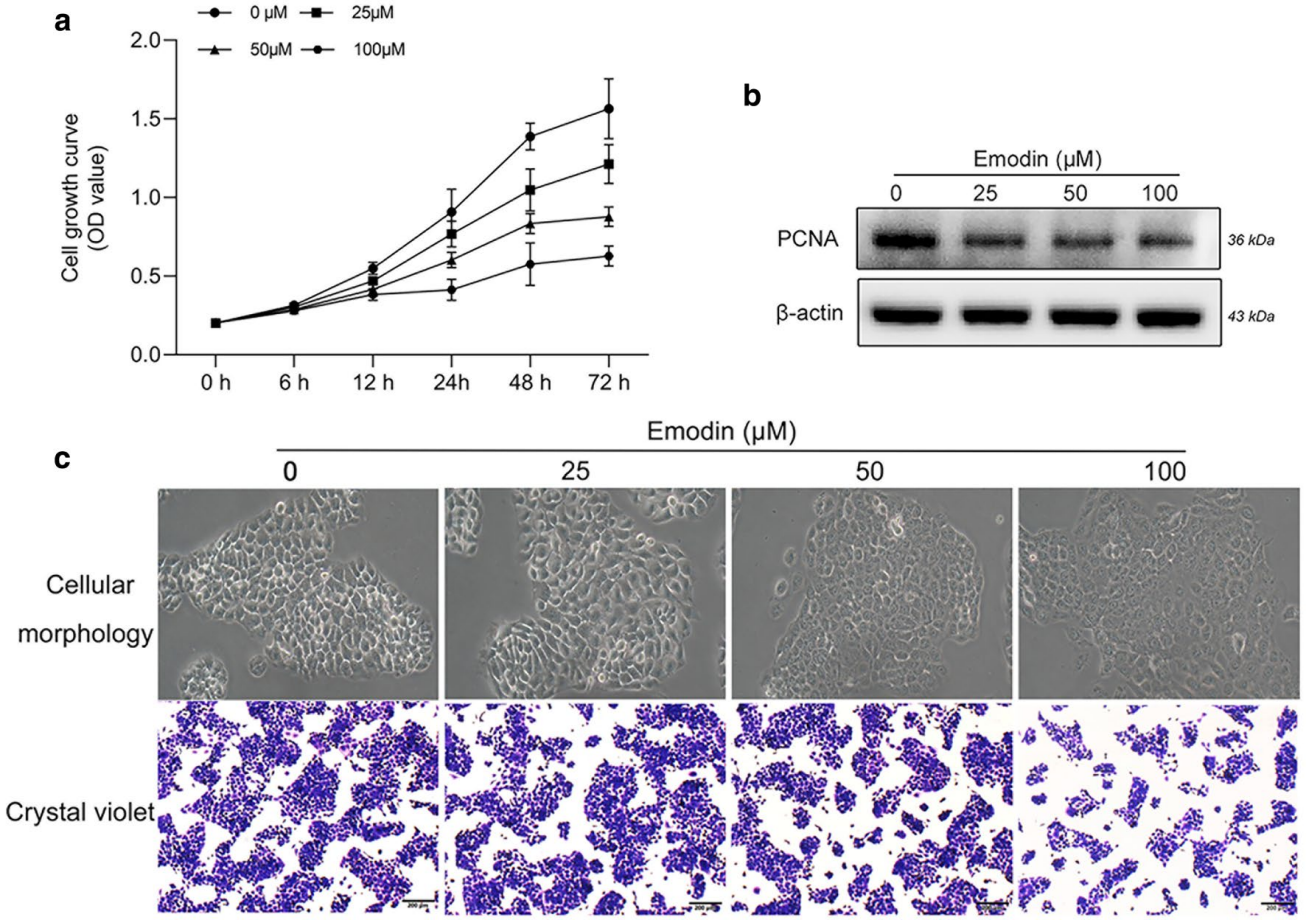
Emodin suppressed the cell proliferation in SZ95 sebocytes. (**a**) Cells were treated with emodin (25, 50 and 100 µM) for 6 to 72 h. The growth curve was determined by CCK-8 assay. (**b**) Upon treating with various concentrations of emodin for 72 h, protein expression of PCNA was measured by western blot. (**c**) Observation of cell morphology under a light microscope (original magnification, ×200), and colony formation was verified by crystal violet staining (scale bar = 200 μm).

To clarify the effect of emodin on the proliferation of cultured SZ95 sebocytes, a CCK-8 assay was performed. The growth rate of sebocytes was significantly reduced upon treatment with emodin at concentrations of 25 to 100 μM, indicating that emodin inhibited sebocyte proliferation in a time-dependent manner (Fig. 1a). In line with the CCK-8 results, we found that the expression levels of proliferating cell nuclear antigen (PCNA), a DNA replication-regulating protein, were markedly decreased in emodin-treated groups (Fig. 1b). The colony-forming activity of SZ95 sebocytes was examined by conducting a low cell seeding assay. Under microscopic observation, the cell morphology exhibited significant shrinkage and disorganized intracellular contents in response to increasing doses of emodin exposure. Based on crystal violet staining, the density of sebocytes was reduced in a dose-dependent manner (Fig. 1c). Collectively, these findings implicated the antiproliferative response elicited by emodin in SZ95 sebocytes.

### Emodin induces G1/S arrest in human SZ95 sebocytes

**Figure 2:**
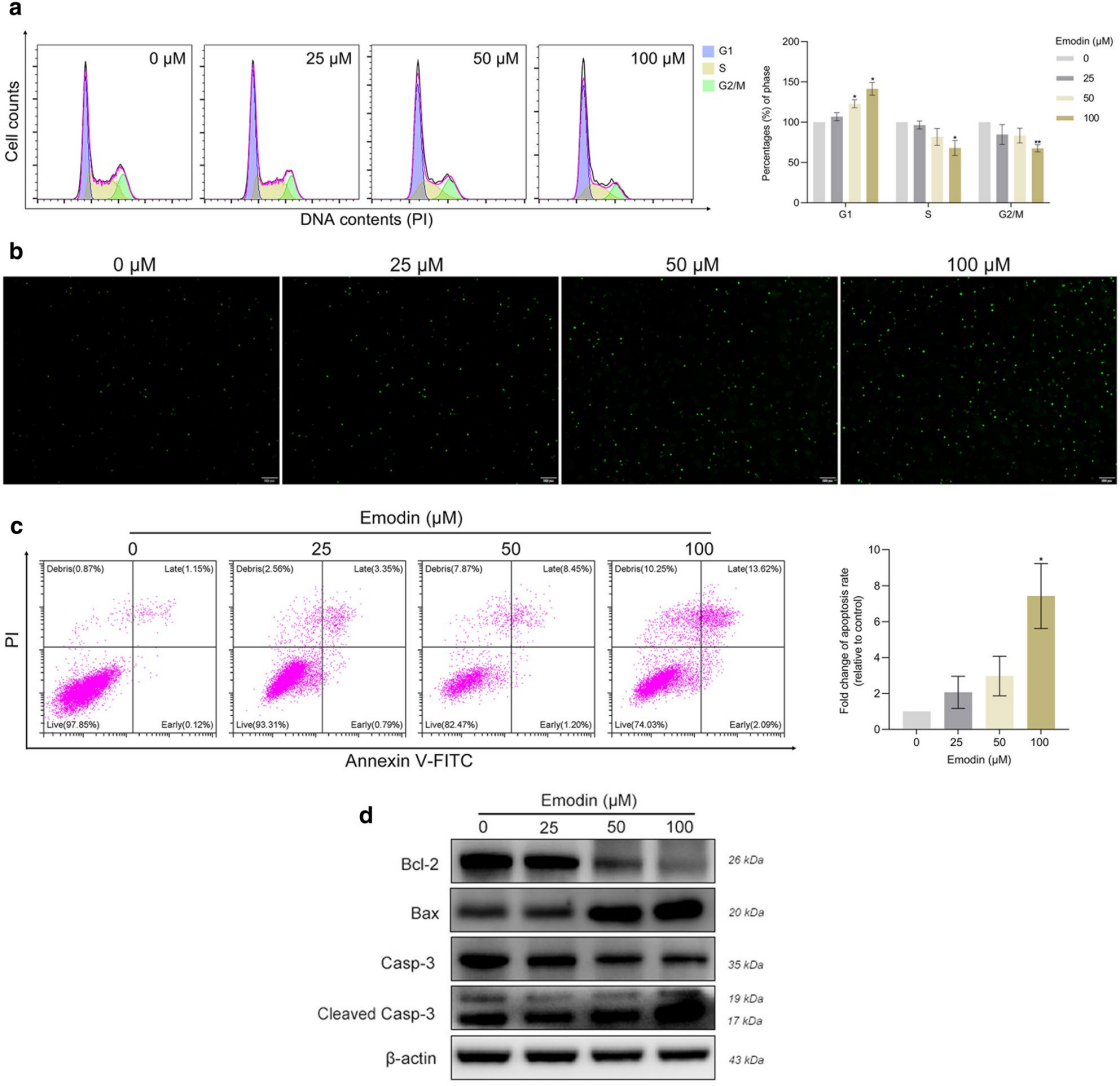
Emodin induced G1/S transition arrest and apoptosis in SZ95 sebocytes. Following treatment with various concentrations of emodin (25, 50 and 100 µM) for 72 h, (**a**) cell cycle phases were determined by flow cytometry. G1, interphase for DNA synthesis; S, DNA synthesis phase; G2/M, mitosis phase. Cell population within different checkpoints, calculated as a percentage of untreated cells. (**b**) TUNEL-positive cells are labeled with green fluorescence. Scale bar = 200 μm. (**c**) Flow cytometric dot plots illustrating the distribution of Annexin V-FITC/PI-stained cells. Bar graphs indicate the relative fold change of apoptotic cells to the untreated cells. (**d**) Protein expression of apoptosis-related proteins (Bcl-2, Bax, caspase-3, cleaved caspase-3) was detected by western blot. Data represent the means ± SDs (n = 3). * *P* < 0.05, ** *P* < 0.01 *vs.* untreated cells. Casp-3, caspase-3; Cleaved Casp-3, cleaved caspase-3.

Flow cytometry was applied to assess whether the antiproliferative effects of emodin are mediated through cell cycle progression. As shown in Fig. 2a, emodin resulted in a dose-dependent increase in the proportion of cells at the G1 phase while decreasing the distribution of cells in the S and G2/M phases. These data suggested that emodin may induce G1 phase arrest in SZ95 sebocytes, thereby disrupting the G1 to S transition and ultimately impeding cell cycle progression.

### Emodin enhances apoptosis in human SZ95 sebocytes

Considering the inhibitory effect of emodin on sebocyte growth, we further determined its role in the manipulation of cell apoptosis. The presence of DNA fragmentation from apoptotic cells was visualized by TUNEL assay. As depicted in Fig. 2b, the prevalence of TUNEL-positive cells was enhanced after treatment with increasing concentrations of emodin. Then, we performed an Annexin V-FITC/PI staining to evaluate the proportion of apoptotic cells. Flow cytometric dot plots indicated that emodin increased the apoptotic cell population in SZ95 sebocytes. In the emodin-treated sebocytes, a significant dose-dependent elevation was found in the percentage of apoptotic cells, including both early and late apoptotic cells (Fig. 2c).

Emodin damaged cell survival in tumor cell lines via apoptotic regulatory proteins, especially the Bcl-2 family^12^. Our data indicated that emodin significantly upregulated the expression of the proapoptotic protein Bax while downregulating the antiapoptotic protein Bcl-2. Additionally, the protein expression of full-length caspase-3 was decreased and cleaved caspase-3 was increased in the emodin-treated cells, as compared to the untreated cells (Fig. 2d). Taken together, these combined results suggested that emodin may play a negative role in sebocyte survival by inducing apoptosis.

### Emodin inhibits lipogenesis of human SZ95 sebocytes

**Figure 3:**
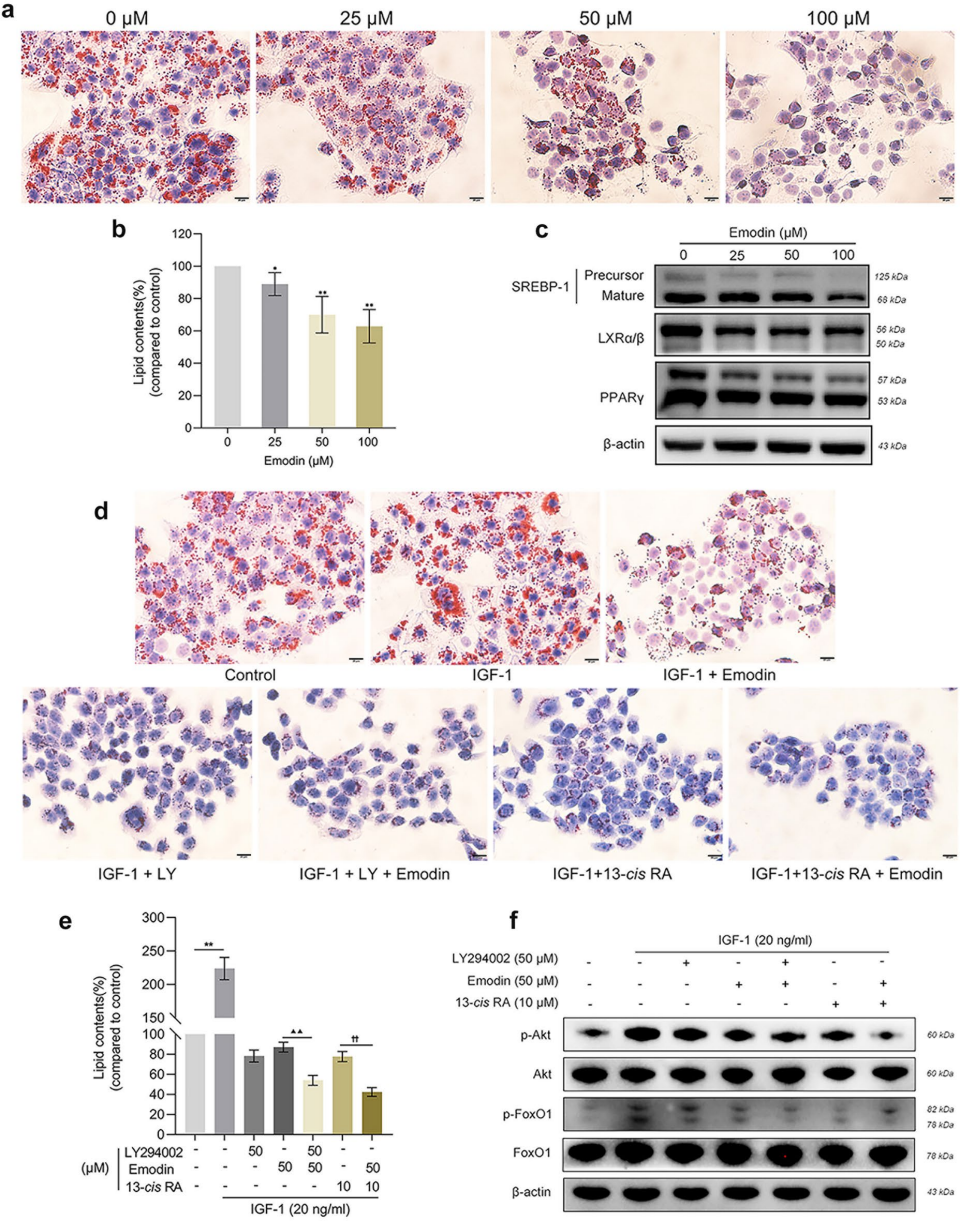
Emodin inhibited lipid accumulation in SZ95 sebocytes. Cells were treated with increasing doses of emodin (25, 50 and 100 µM) for 24 h. (**a**) Intracellular lipids were visualized by ORO staining. Scale bar = 20 μm. (**b**) The OD value of the supernatant ORO contents was spectrophotometrically quantified at OD_500_ and calculated as percentages relative to untreated cells. (**c**) Lipogenic proteins (PPARγ, LXRα/β and SREBP-1) were detected by western blot. SZ95 sebocytes were pretreated with IGF-1 (20 ng/ml) for 6 h, followed by exposure to PI3K inhibitor LY294002 (50 μM) or 13-*cis* RA (10 μM) in the presence or absence of emodin (50 μM) for 24 h. (**d**) Lipid droplets were photographed with ORO staining. Scale bar = 20 μm. (**e**) Lipid levels were quantitated at OD_500_ and determined as percentages to untreated sebocytes. (**f**) Protein expression of p-Akt, Akt, p-FoxO1 and FoxO1 was assessed by western blot. Data represent the mean ± SD (n = 3). *P < 0.05, **P < 0.01 *vs*. untreated cells; ^▲▲^*P* < 0.01 *vs* IGF-1 + LY294002 group; ^††^*P* < 0.01 *vs* IGF-1 + 13-*cis* RA group. p-, phosphorylated; LY, LY294002.

The primary function of sebaceous cells is to synthesize and then secrete lipid-rich sebum. To investigate the impact of emodin on sebaceous lipogenesis, we examined the production of neutral lipid profile using Oil Red O staining, and found that emodin exerted a dose‐dependent lipogenesis‐reducing activity in SZ95 sebocytes (Fig. 3a,b). To provide molecular evidence for emodin-mediated sebosuppressive effect, we examined the protein expression of lipogenic transcription factors by western blot. PPARγ, SREBP-1 and LXRs are key genes implicated in sebaceous lipogenesis and differentiation^5^. Our results indicated that emodin markedly decreased the protein expression of PPARγ, LXRα/β and SREBP-1 in a dose-dependent manner (Fig. 3c), supporting the notion that emodin can suppress the differentiation and sebum synthesis in SZ95 sebocytes.

### Emodin inactivates the PI3K/Akt/FoxO1 signaling pathway in IGF-1-treated SZ95 sebocytes

To clarify the effects of emodin in acne‐mimicking lipid synthesis, we induced sebocyte differentiation and lipogenesis by stimulating with 20 ng/ml IGF-1. IGF-1-treated sebocytes exhibited an increase in both cellular size and lipid accumulation, as indicated by Oil red O staining (Fig. 3d,e). Lipid overproduction induced by IGF-1 was significantly reversed by treatment with emodin. IGF-1 has been proposed as an PI3K agonist and promotes sebaceous lipogenesis by activating the downstream Akt/FoxO1 pathway^6^. As shown in Fig. 3f, the protein expression of p-Akt and p-FoxO1 were increased by IGF-1 and downregulated by PI3K inhibitor LY294002. Treatment with emodin further inhibited the phosphorylation of Akt and FoxO1, while having no impact on total protein expression of Akt and FoxO1 in SZ95 sebocytes under the intervention of LY294002. Overall, emodin inhibited the activation of the PI3K/Akt/FoxO1 signaling pathway induced by IGF-1, which demonstrated the specific inhibitory effect of emodin on the PI3K transduction.

13-*cis* RA, the most effective anti-acne drug, suppresses sebaceous glands activity and normalizes the infundibular hyperkeratinization^10^. Our previous study revealed that 13-*cis* RA dose-dependently inhibited the phosphorylation of FoxO1 in human primary keratinocytes^17^. We then examined the combined effects of 13-*cis* RA and emodin on PI3K/Akt/FoxO1 pathway in sebocytes. Firstly, by using CCK-8 assay, we found that up to 10 µM 13-*cis* RA did not decrease the cell viability of SZ95 sebocytes (Supplementary Fig. S1). Co-treatment with 13-*cis* RA reduced the lipid droplet accumulation and neutral lipid contents in SZ95 sebocytes compared to those exposed to IGF-1, as revealed by ORO lipid staining. Western blot demonstrated a reduction in the phosphorylated levels of Akt and FoxO1 when treated with 13-*cis* RA or emodin. After the combined administration of 13-*cis* RA and emodin, the protein expression of p-Akt, p-FoxO1 were significantly decreased in sebocytes treated with IGF-1, which definitely illustrated the ability of emodin to enhance the anti-IGF-1 effects exerted by 13-*cis* RA (Fig. 3d,f). Above all, these results illuminated that emodin inhibits the activation of the PI3K/Akt/FoxO1 pathway in human sebocytes, suggesting that emodin may stagnate acne formation by inhibiting IGF-1-induced lipogenesis.

### Anti-inflammatory effects of emodin are mediated by inhibiting the NLRP3 signaling in *C. acnes*-treated SZ95 sebocytes

**Figure 4:**
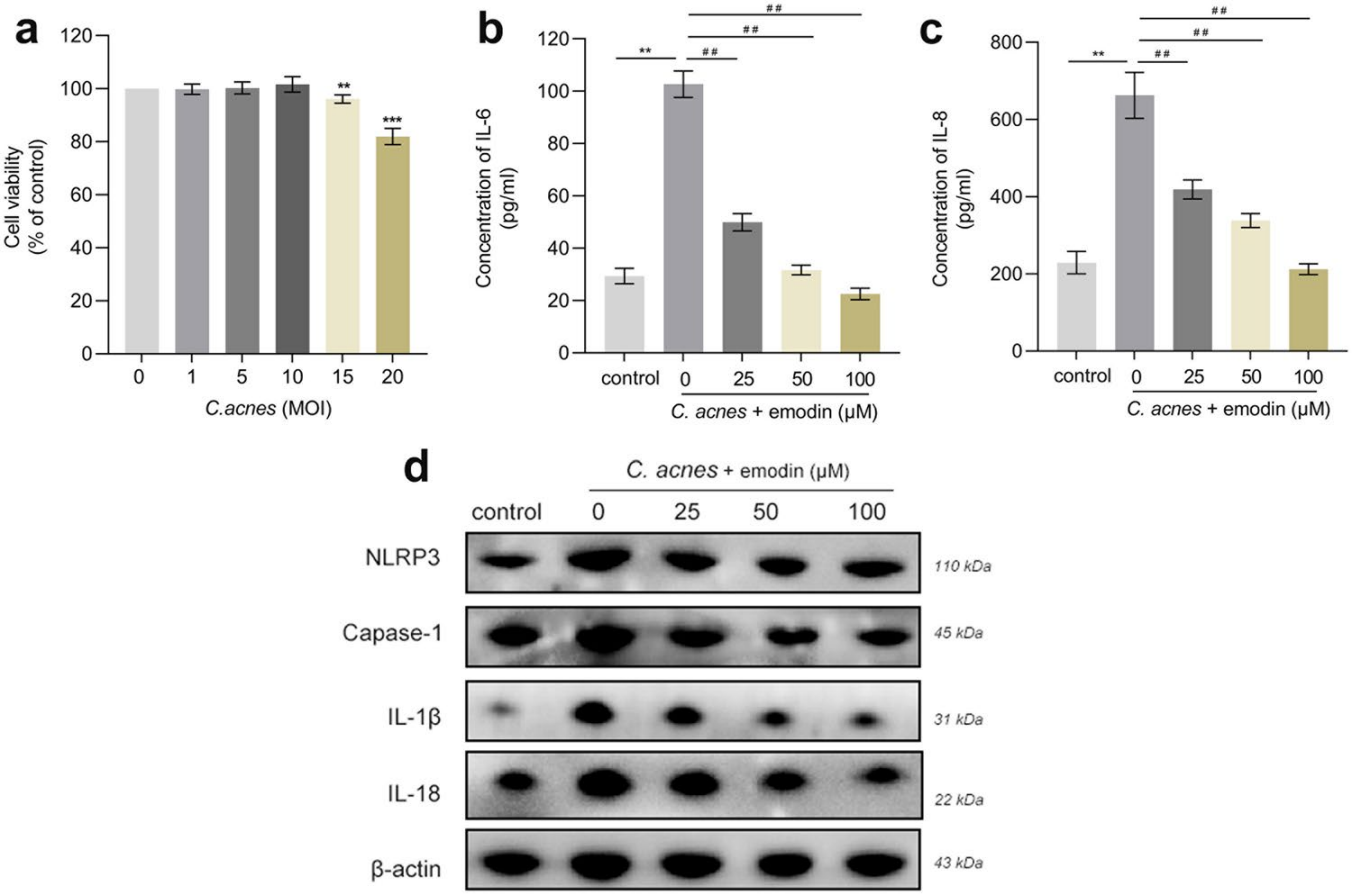
Emodin ameliorated *C. acnes*-stimulated inflammation by inhibiting the NLRP3 inflammasome in SZ95 sebocytes. (**a**) Cells were incubated with various doses of *C. acnes* (1 to 20 MOI) for 6 h and then cell viability was assessed by CCK-8 assay. (**b**,**c**) ELISA was performed for cytokine production of IL-6 and IL-8 under the treatment of emodin (25, 50 and 100 µM) after stimulation by *C. acnes* (10 MOI). (**d**) The expression of NLRP3, caspase-1, IL-1β and IL-18 was detected by western blot. Data are presented as the means ± SDs (n = 3). *P < 0.05, **P < 0.01 *vs*. control. ^#^*P* < 0.05, ^##^*P* < 0.01, ^###^*P* < 0.001 *vs*. *C. acnes*-treated sebocytes.

*Cutibacterium acnes* stimulates sebocytes to produce interleukin (IL)-6 and IL-8 and other cytokines, thereby aggravating inflammatory responses in acne progression^18^. Accordingly, we further investigated the impact of emodin on *C. acnes*-induced inflammation in SZ95 sebocytes. The viability of sebocytes coincubated with *C. acnes* was evaluated using the CCK-8 assay (Fig. 4a). We found that the viability of SZ95 sebocytes was not impaired by *C.* *acnes* at concentrations up to 10 MOI after a 6-h exposure. Therefore, the in-vitro inflammation model was elicited by 10 MOI of *C. acnes*, as we previously described^19^. As anticipated, ELISA measurement revealed that *C. acnes* induced an upregulation of IL-6 and IL-8, while the production of the above cytokines was significantly decreased in emodin-treated SZ95 sebocytes (Fig. 4b,c).

The activation of NLRP3 inflammasome by *C. acnes* leads to the secretion of pro-inflammatory cytokines in various immune cells^20^. We next monitored whether emodin regulates this complex in sebocytes. Our data showed that *C. acnes* stimulation induced an upregulation of proteins involved in the NLRP3 inflammasome, including NLPR3, caspase-1, IL-1β and IL-18 expression (Fig. 4d). However, these effects were markedly reversed by emodin treatment in a dose-dependent manner. Therefore, our results provide evidence that emodin possesses anti-inflammatory activity against the immune response induced by *C. acnes* in SZ95 sebocytes.

## Discussion

Anthraquinones, a group of secondary metabolites of herbs, have been shown to target comedogenic processes. Chrysophanol, extracts of the seeds in *Cassia tora*, suppresses lipid synthesis and inflammation induced by heat exposure in human sebocytes^21^. Rhein, an active substance of rhubarb, inhibits the growth of *C. acnes*^22^. Physcion from *Polygonum multiflorum*, is a potent inhibitor of 5α-reductase by reducing hair follicle sensitivity to androgen—a lipogenic factor capable of facilitaing sebaceous growth and lipogenesis^23^. However, the potential of emodin in treating acne remains largely unknown.

The active regeneration of sebocytes during puberty leads to excessive lipid storage, which promotes the development of acne. Therefore, effective therapy for adolescent acne would involve the prevention of the aberrant proliferation status in SGs. Emodin has garnered attention for its effectiveness in treating hyperproliferative skin diseases by impairing cellular viability and promoting cell death in human keratinocytes, melanoma cells and squamous cell carcinoma cells^24^. PCNA is a crucial factor for DNA replication, and emodin can inhibit tumor cell proliferation and invasion through a PCNA-dependent mechanism^25^. Our results suggest that emodin reduced cell viability and colony formation by downregulating the PCNA expression in SZ95 sebocytes.

Mounting evidence supports the ability of emodin to alter cell cycle distribution, while its effects may vary depending on cell types. Emodin induces G2/M arrest in the hepatocellular carcinoma cell lines, while casuing G0/G1 or sub-G1 arrest in gastric cancer cells^12^. Consistent with prior investigations in cancer cells, we found that emodin was capable of arresting sebocytes in the G1 to S transition. Interestingly, PCNA can form a complex with the p21 protein. DNA damage increases p21 production, which subsequently leads to the reduced level of PCNA and halts the transition from G1 to S phase^26^. These studies not only better explain the reduction in colony formation observed in emodin-treated sebocytes, but also indicate that emodin may inhibit the proliferation by PCNA-dependent cell cycle arrest.

The members of Bcl-2 family are typically divided into two subgroups, including antiapoptotic proteins (e.g., Bcl-2) and proapoptotic proteins (e.g., Bax)^27^. Notably, Bax induces the translocation of cytochrome-c (cyt-c) from the mitochondria into the cytoplasm, thereby initiating caspase-9 activation. Once activated by caspase-9, the caspase-3 protein cleaves into two subunits and subsequently functions as an executioner to trigger cell apoptosis^28^. Bax can be functionally inhibited by dimerizing with Bcl-2, which impedes the release of mitochondrial cyt-c and halts caspase-3 activation^29^. Emodin is a powerful inducer of apoptosis by regulating the activity of the Bcl-2 family and caspase cysteine proteases. Our study found that Bcl-2 expression was significantly downregulated, whereas Bax and cleavage caspase-3 increased. We speculate that emodin-induced apoptosis may be associated with the activation of mitochondrial death signals.

In SGs, lipid synthesis is under the control of differential and lipogenic factors. PPARγ and its agonists have been shown to enhance differentiation and lipogenesis in human sebocytes^30^. Both LXR and its target SREBP-1 are core regulators of cholesterol homeostasis and lipid metabolism^4^. In adipose tissue, emodin can alleviate fat accumulation by inhibiting adipogenic differentiation^14^. Our data highlighted that emodin dramatically downregulated the expression of PPARγ, LXR and SREBP-1 in SZ95 sebocytes. FoxO1 decreases serum synthesis by inhibiting the transcriptional activity of PPARγ, LXR and SREBP-1. However, exogenous IGF-1 improves the activation of PI3K/Akt signaling, which results in FoxO1 degradation by post‑translational modification of phosphorylation^6^. We found that emodin significantly reduced IGF-1-induced lipid overproduction and the phosphorylated levels of Akt and FoxO1. Thus, emodin may counteract the pro-acne effects of IGF-1 by inhibiting the phosphorylation of PI3K/Akt/FoxO1 pathway, thereby downregulating the expression of lipogenic factors. In addition, FoxO1 is also a key target involved in 13-*cis* RA-induced apoptosis^31^. We observed a significant synergy between 13-*cis* RA and emodin in suppressing IGF-1-stimulated lipogenesis and phosphorylation of Akt and FoxO1 in SZ95 sebocytes.

The activation of the NLRP3 inflammasome promotes the maturation of caspase-1 and triggers the release of the downstream acne-pathogenic cytokines IL-1β and IL-18, which subsequently activates the secondary inflammatory mediators, including IL-6 and IL-8^32^. However, the inhibition of this complex can alleviate the inflammatory responses mediated by *C. acnes* in human sebocytes^19^. The anti-inflammatory properties of emodin have been extensively documented in various cells via the NLRP3 inflammasome signaling^33^. In our present study, C. *acnes* stimulated the overexpression of NLRP3, caspase-1, IL-18, IL-1β, IL-6 and IL-8, which was markedly attenuated following emodin treatment in SZ95 sebocytes. These results are in alignment with previous reports that emodin may inhibit the activation of the NLRP3 inflammasome. Since emodin also targets other pattern recognition receptors, such as Toll-like receptors^34^, further investigations are required to investigate whether emodin would exert its inhibitory effect on *C. acnes*-induced inflammation through these alternative mediators.

The development of acne is a composite of multifarious pathological processes. During the acneigenic stage, abnormal hyperproliferation of follicular keratinocyte and excessive sebum secretion by SGs are primarily responsible for microcomedo formation, which simultaneously facilitate *C. acnes* colonization and *C. acnes*-derived inflammatory cascade^8^. A previous study revealed that emodin exerted a pro-apoptotic effect on HaCaT keratinocytes^24^. Our supplemental data suggested that treatment with emodin suppressed the proliferation of HaCaT keratinocytes. The lipids produced by keratinocytes are one of the main components of the skin lipid profiles on human epidermis, some of which are catalyzed by the lipid synthases to generate sebum in sebocytes^35^. IGF-1 increased intracellular lipid accumulation in HaCaT keratinocytes, which was significantly inhibited when treatment with emodin. In addition, emodin alleviated *C. acnes*-induced inflammatory cytokines secretion, including IL-6, IL-8 and TNF-α in HaCaT keratinocytes (Supplementary Fig. S2). These findings demonstrated that treatment with emodin may be a therapeutic approach for addressing keratinocytes-mediated skin dysfunction in acne. Also, the evaluation of acne prognosis, particularly in relation to atrophic scars, heavily relies on the bioactivity of dermal fibroblasts in collagen production^36^. It has been reported that emodin downregulates the protein expression of matrix metalloproteinases (MMP)-2/-9 in melanoma cell lines^37^, but it is still unclear about the effect of emodin on maintaining the equilibrium between MMPs and fibrosis in skin fibroblasts. Further investigations are needed to clarify it.

## Conclusions

**Figure 5:**
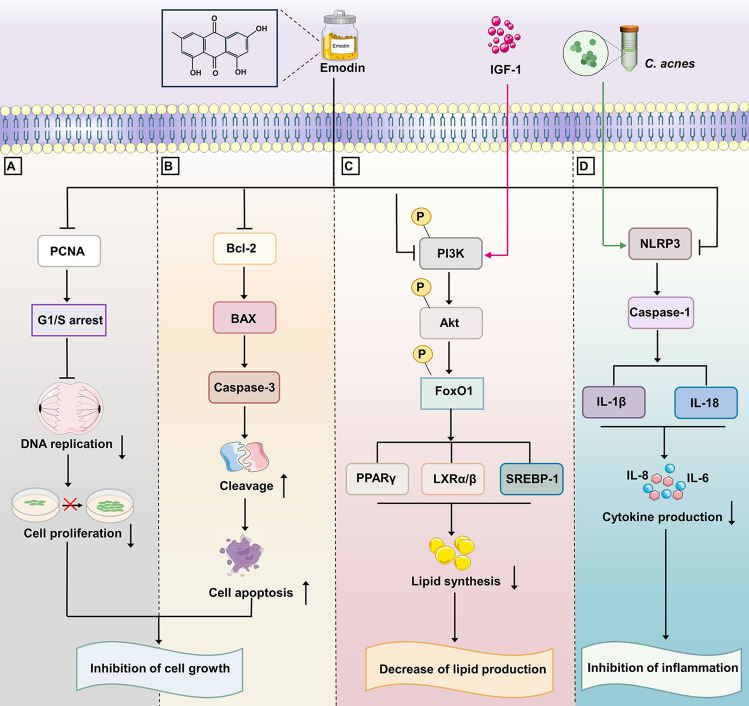
Effects of emodin on acne-related pathological processes in SZ95 sebocytes. (**A**) Inhibition on cell proliferation by arresting the cell cycle in a PCNA-dependent manner. (**B**) Induction of apoptosis by downregulating Bcl-2 and upregulating Bax and caspase-3 cleavage. (**C**) Inhibition on IGF-1-induced lipogenesis by inhibiting the PI3K/Akt/FoxO1 pathway. (**D**) The inactivation of the NLRP3 inflammasome mitigates the production of pro-inflammatory cytokines IL-1β and IL-18 stimulated by *C. acnes*.

In conclusion, our results initially demonstrated that emodin can suppress proliferation, induce cell cycle arrest and apoptosis, inhibit lipogenesis and inflammation in human SZ95 sebocytes (Fig. 5). We found that IGF-1 promoted lipid production, whereas emodin significantly inhibited this effect by inactivating the PI3K/Akt/FoxO1 pathway. Moreover, emodin alleviated *C. acnes*-stimulated pro-inflammatory cytokines production and inactivated the NLRP3 inflammasome. Hence, our present study provides a novel perspective into the therapeutic potential involved in the emodin treatment for acne.

## Materials and methods

### Cell culture and pharmacological treatment

Human immortalized SZ95 sebaceous gland cells were kindly granted from Prof. Zouboulis^38^. In brief, SZ95 sebocytes were established by the retroviral vector pSVT, which contains the sequences encoding the SV-40 large T protein for the transformation of primary sebocytes derived from human sebaceous glands. SZ95 sebocytes were maintained in Sebomed basal medium (Sigma-Aldrich, MO, USA) containing 10% fetal bovine serum (Gibco, Rockville, MD) and 5 ng/ml recombinant human epidermal growth factor (Invitrogen, Carlsbad, CA) at 37 °C in a humidified incubator with 5% CO_2_. The pharmacological reagents, including emodin, IGF-1, LY294002 and 13-*cis* RA, were dissolved in dimethyl sulfoxide (DMSO). The final concentration of DMSO in the culture medium during the experiments was maintained below 0.1%.

### Preparation of *C. acnes*

The *C. acnes* strain (ATCC#6919) was obtained from Guangdong Microbial Culture Collection Center (Guangzhou, China). *C. acnes* was cultured in brain–heart infusion agar (Oxoid, Basingstoke, England) until reaching stationary phase at 37 °C under anaerobic conditions. Bacterial cultures were blended until the OD_600_ reached approximately 1.0 and harvested via centrifugation at 12,000×*g* for 5 min and resuspended in PBS. The pellets were diluted to achieve final concentrations of 1, 5, 10, 15 and 20 multiplicity of infection (MOI). The suspension of *C. acnes* was stored at 4 °C for further experiments.

### In vitro acne models

To induce cell differentiation, SZ95 sebocytes were pretreated with IGF-1 (20 ng/ml) for 6 h. Then, cells were exposed to the PI3K inhibitor LY294002 (50 μM) or 13-*cis* RA (10 μM) in the presence or absence of emodin (50 μM) for 24 h. Alternatively, to imitate the acne-prone inflammatory micro-environment, *C. acnes* (10 MOI) was incubated with sebocytes for 6 h. Culture media were replaced with fresh media and cells were further treated with various doses of emodin (25, 50 and 100 μM) for 24 h.

### CCK-8 assay and crystal violet staining

CCK-8 assay was used for cell proliferation detection. SZ95 sebocytes (2000 cells per well) were seeded on 96-well plates and cultured with varying concentrations of emodin for 6 to 72 h. At the designated timepoint, cells were replenished with fresh medium containing 10 μl of CCK-8 solution and further incubated for 40 min. The optical density (OD) was measured at 500 nm using a Multiskan GO microplate multimode reader (Thermo Fisher Scientific). For colony formation detection, SZ95 sebocytes (1 × 10^4^ cells per well) were seeded on 6-well plates and treated with emodin for 72 h. Cells were stained with crystal violet for 10 min and imaged under a phase-contrast microscope (Olympus Corporation, Tokyo, Japan).

### Flow cytometry

For cell cycle and apoptosis detection, cells stained with PI and Annexin V-FITC were analyzed with a flow cytometer (Beckman Coulter, Fullerton, CA, USA). The cell cycle is within G1, S, and G2/M phases. The cell population is divided into four groups: live cells as Annexin V-FITC and PI staining both negative; cellular debris as Annexin V-FITC negative and PI positive; early-apoptotic cells as Annexin V-ITC positive and PI negative; late-apoptotic cells as Annexin V-FITC and PI both stain positive. Data were measured using FlowJo.10.8.1 software.

### TUNEL staining

SZ95 sebocytes were cultured in 24-well plates and treated with increasing doses of emodin. Cells were fixed with 4% formaldehyde for 30 min and permeabilized with 0.3% Triton X-100 in PBS for 10 min. Then, the samples were incubated with TUNEL solution for 1 h in the dark and visualized by a BX51 fluorescence microscope (Olympus Corporation, Tokyo, Japan).

### Oil red O (ORO) staining

When reaching 50% confluency, SZ95 sebocytes were treated with emodin for 24 h. Cells were fixed with 4% formaldehyde at RT for 10 min. Prior to usage, a mixture of ORO stock solution (0.5% in 99% isopropanol) and double distilled H_2_O were filtered through a 0.22 μm sterile syringe filter at a ratio of 6:4. After being stained with freshly prepared ORO for 30 min, cells were washed with PBS twice, counterstained with hematoxylin and then observed by light microscopy (Olympus Corporation, Tokyo, Japan). For lipid extraction, ORO-stained cells were incubated with isopropanol for 10 min, and the optical density (OD) of extracted ORO content in the supernatant was measured at 500 nm using microplate reader (Thermo Fisher Scientific, Rockford, IL, USA).

### Western blot

Cells were lysed in PRO-PREP protein extraction solution (Intron, Daejeon, Korea). Proteins were centrifuged at 13,201×*g* for 10 min at 4 °C, and their concentrations were assessed by BCA assays. A total of 20 μg protein was loaded on SDS–polyacrylamide gels and electro-transferred onto PVDF membranes. Membranes were blocked with non-fat milk for 1 h, and incubated overnight at 4 °C with following primary antibodies: PCNA, Bcl-2, Bax, caspase-3, cleaved caspase-3, PPARγ, SREBP1, LXRα/β, p-Akt, Akt, p-FoxO1, FoxO1, NLRP3, caspase-1, IL-1β, IL-18 and β-actin (1:1000). Blots were incubated with peroxidase-conjugated secondary antibodies (1:5000; Santa Cruz Biotechnology) and visualized by enhanced chemiluminescence. Original blots have been presented in Supplementary Information.

### Enzyme-linked immunosorbent assay (ELISA)

Following the stimulation of *C. acnes*, subsequent cultivations were carried out with emodin for 24 h. To quantify cytokine production, the culture supernatants were collected and analyzed for levels of IL-8 and IL-6 using ELISA kits (Solarbio, Beijing, China) according to the manufacturer’s protocols.

### Statistical analysis

Statistical analysis was performed using GraphPad Prism 9.0 software. All experiments were repeated at least three times with different batches. Data are presented as the mean ± standard deviation (SD). Comparisons between two groups and multiple groups were analyzed by Student's t-test and One-way ANOVA, respectively. *P* < 0.05 were considered statistically significant.

### Supplementary Information

Supplementary Figures.

## Data Availability

Requests for data and materials should be addressed to G.S.
