# Supplementary material for: Emodin exhibits anti-acne potential by inhibiting cell growth, lipogenesis, and inflammation in human SZ95 sebocytes
Source: Sci Rep. 2023 Dec 7;13:21576. doi: 10.1038/s41598-023-48709-x (PMC10703917; doi:10.1038/s41598-023-48709-x)
Supplement: Supplementary file 1 — Supplementary Figures. [file 41598_2023_48709_MOESM1_ESM.pdf]

Supplementary Information

Emodin exhibits anti-acne potential by inhibiting cell growth, lipogenesis, and inflammation in human SZ95 sebocytes

Si Liu[1, 2], Xiao-Hua Luo[1, 2], Yu-Feng Liu[1, 2], Christos C. Zouboulis [3] & Ge Shi [1, 2]

[1]Department of Cosmetic and Plastic Surgery, the Sixth Affiliated Hospital, Sun Yat-sen University, Guangzhou, China.

[2]Biomedical Innovation Center, the Sixth Affiliated Hospital, Sun Yat-sen University, Guangzhou, China.

[3]Departments of Dermatology, Venereology, Allergology and Immunology, Staedtisches Klinikum Dessau, Brandenburg Medical School Theodor Fontane and Faculty of Health Sciences Brandenburg, Dessau, Germany.

Supplementary Methods S1.

Reagents

Cell Counting Kit-8 (CCK-8) and Annexin V-FITC/ Propidium Iodide (PI) double staining kit were purchased from Dojindo (Shanghai, China). The crystal violet and TUNNEL assay were purchased from (Beyotime, Shanghai, China). Reagents including emodin, IGF-1 and 13-*cis* RA were obtained from Sigma-Aldrich (St. Louis, MO, USA). LY294002 was purchased from Cell Signaling Technology (Danvers, MA, USA). Cell Cycle Analysis Kit was purchased from Yeasen Co. (Shanghai, China). Antibodies against PCNA(#7907), SREBP-1(17755), LXRα/β (#271064) and β-actin (#47778) were purchased from Santa Cruz Biotechnology (CA, USA); PPARγ (#2435), caspase-3 (#9662), cleaved caspase-3 (#9664), Bcl-2 (#15071), Bcl-2-Associated X (BAX) (#14796), p-Akt (#4060), Akt (#9272), p-FoxO1 (#9464), FoxO1 (#2880), NLRP3 (#15101), caspase-1 (#98033), IL-18 (#67775S) and IL-1β (#12703) were purchased from Cell Signaling Technology (Danvers, MA, USA).

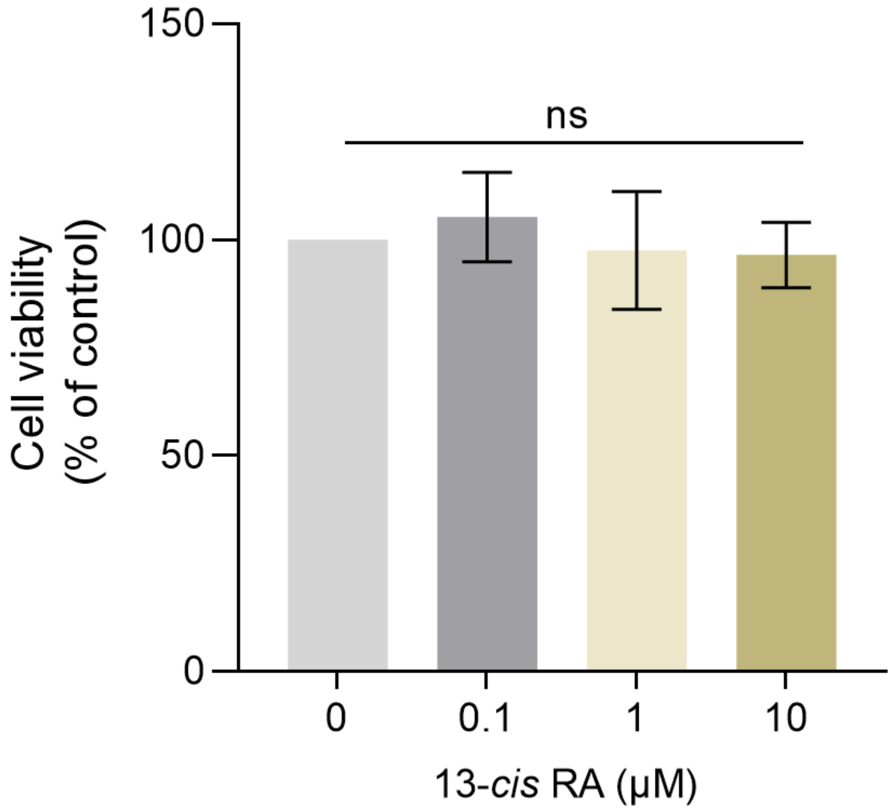

Supplementary Figure S1. Cell viability of SZ95 sebocytes exposed to various concentrations of 13-*cis* RA. SZ95 sebocytes were pretreated with 13-*cis* RA (0.1, 1 and 10 μM) for 24 h and then cell viability was examined by CCK-8 assay. Data represent the means ± SDs (n= 6). No significance (ns) *vs.* untreated cells.

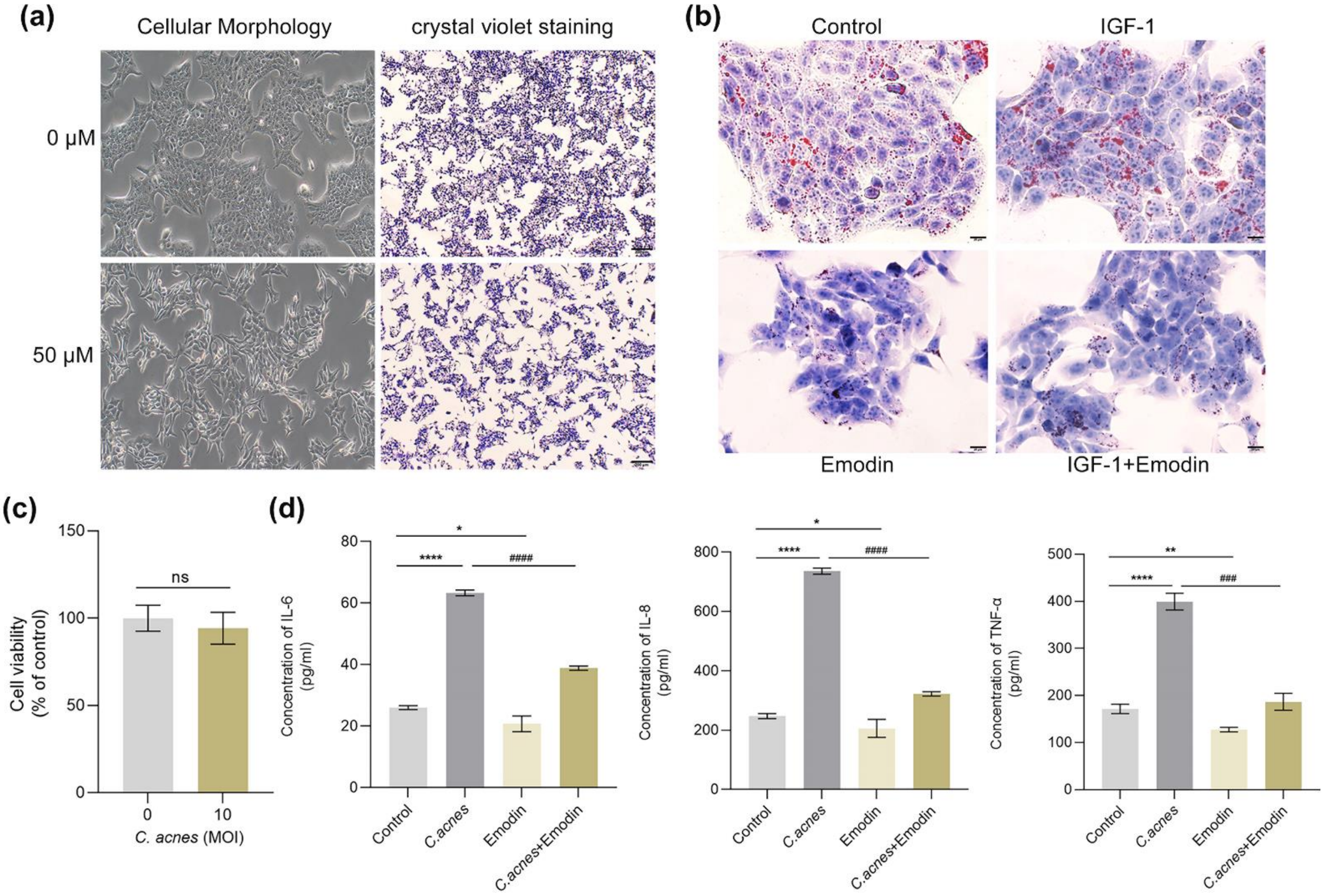

Supplementary Figure S2. Emodin suppressed proliferation, inhibited lipogenesis and reduced inflammatory cytokines secretion in HaCaT keratinocytes. (a) HaCaT keratinocytes (1×10^4 cells per well) were seeded on 6-well plates. After overnight incubation, cells were treated with emodin (50 μM) for 72 h. The morphology of the HaCaT keratinocytes was observed under a light microscope (original magnification, ×100), and then cells were stained with crystal violet for colony formation detection, scale bar = 200 μm. **(b)** SZ95 sebocytes were stimulated with IGF-1 (20 ng/ml) for 6 h, followed by incubation with emodin (50 μM) for 24 h. Lipid droplets were verified by Oil Red O staining. Scale bar = 20 μm. **(c)** After treating cells with *C. acnes* (10 MOI) for 6 h, cell viability was measured by CCK-8 assay**. (d)** ELISA was performed for cytokine production of IL-6, IL-8 and TNF-α under the treatment of emodin (50 μM) after stimulation by *C. acnes* (10 MOI). No significance (ns), and **** $P < 0.0001$ *vs.* untreated cells. $^{###}$ $P < 0.001$, $^{####}$ $P < 0.0001$ *vs*. cells treated with *C. acnes*.

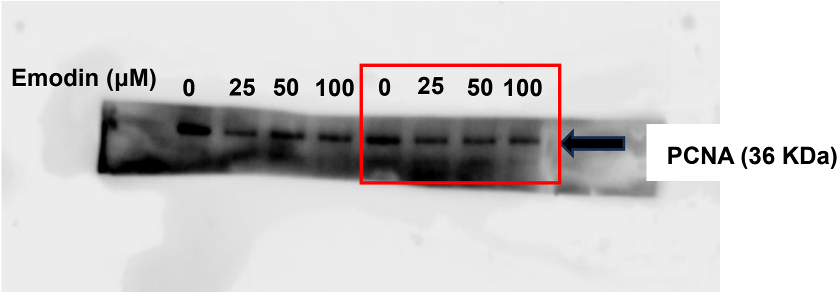

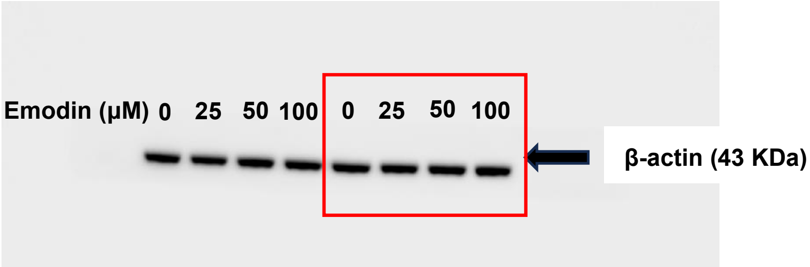

Supplementary Figure S3. Uncropped blots for Figure 1b.

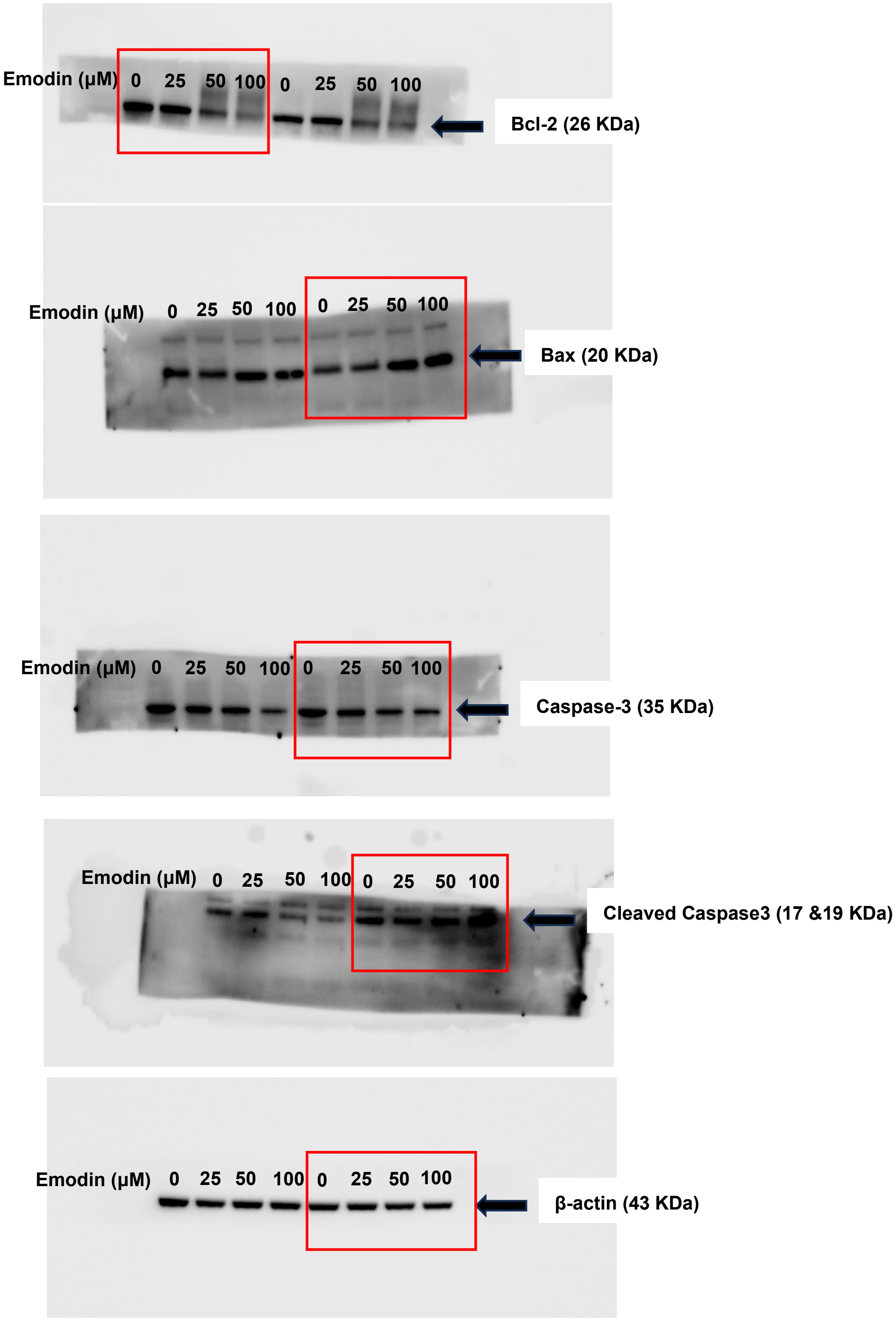

Supplementary Figure S4. Uncropped blots for Figure 2d.

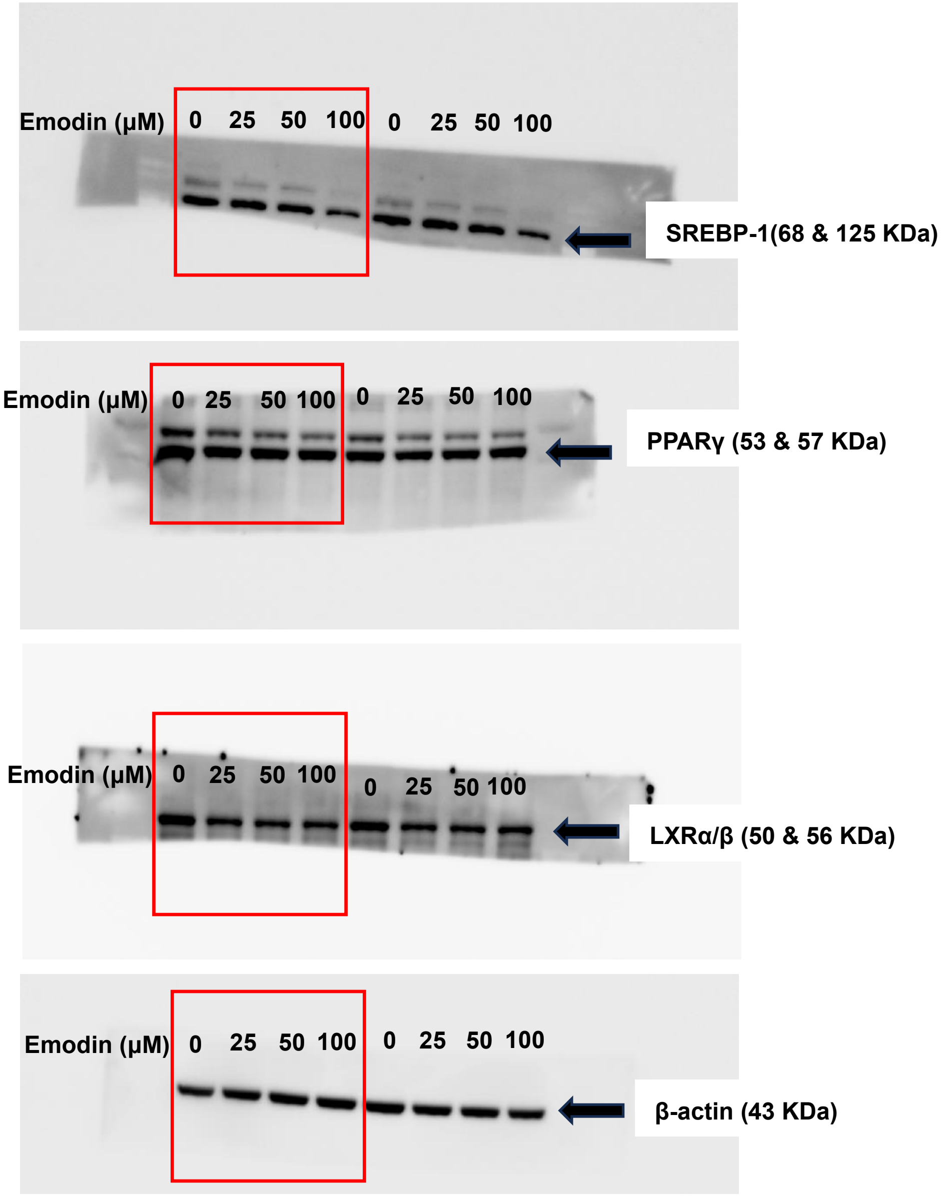

Supplementary Figure S5. Uncropped blots for Figure 3c.

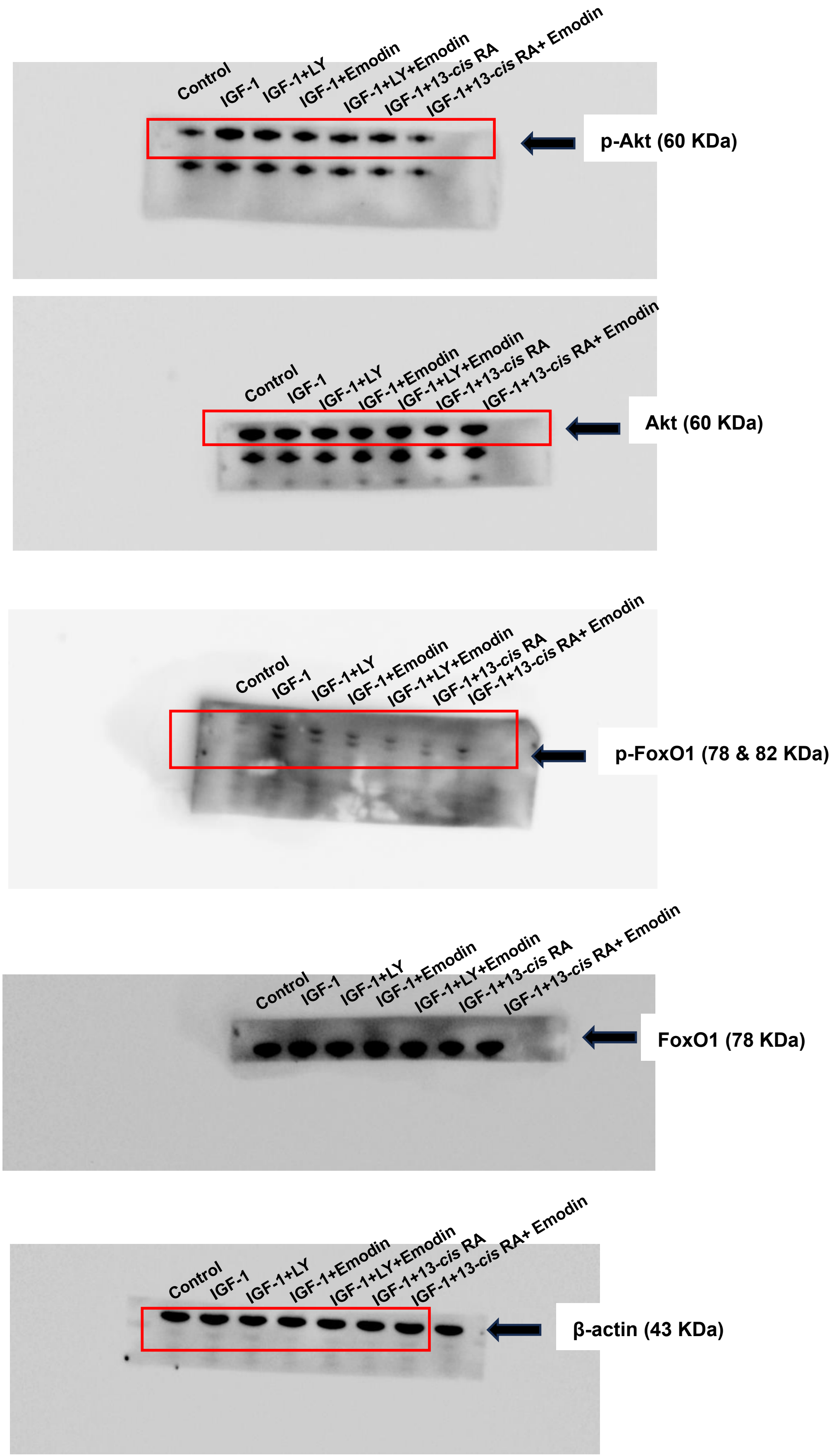

Supplementary Figure S6. Uncropped blots for Figure 3f.

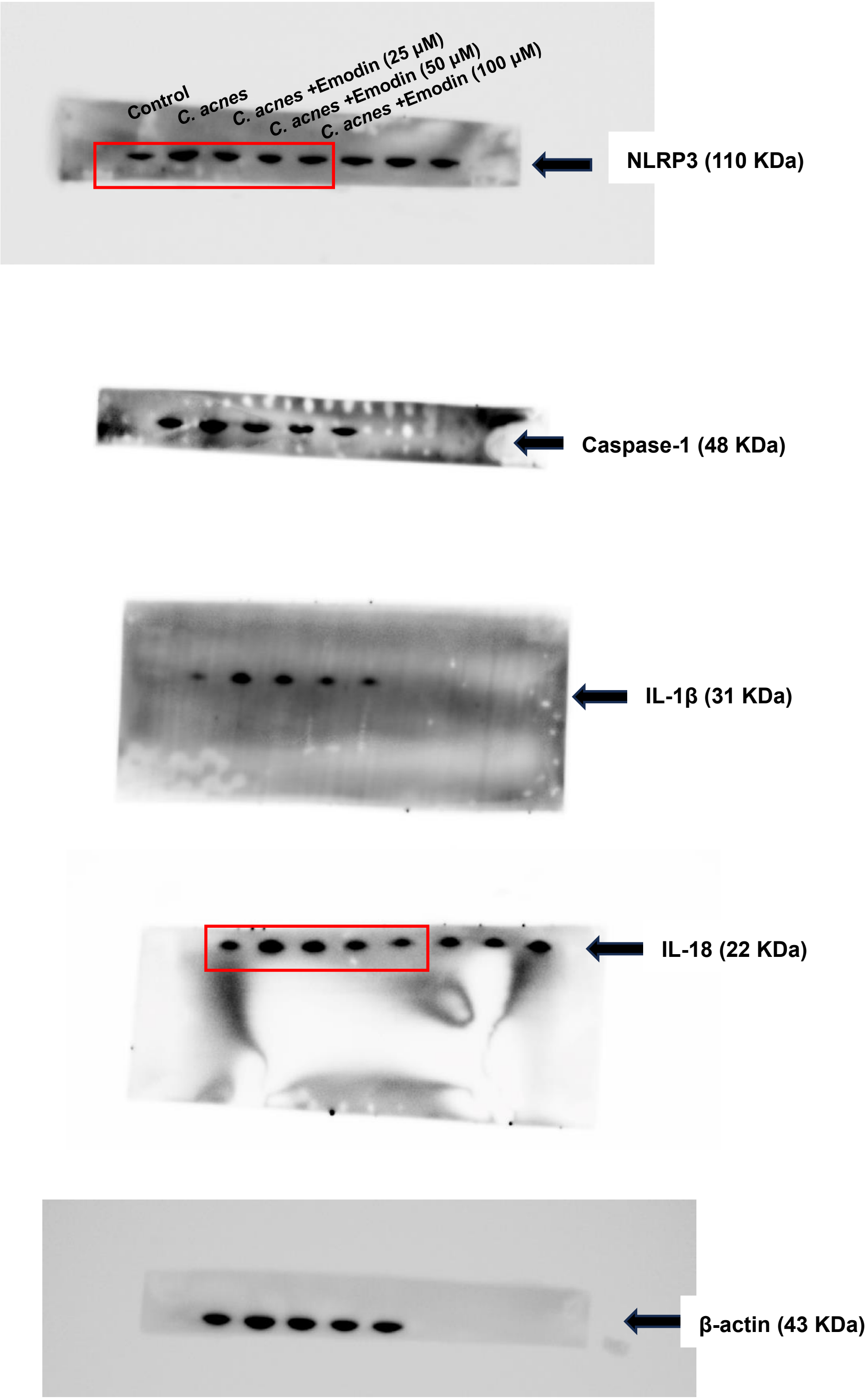

Supplementary Figure S7. Uncropped blots for Figure 4d.

(a) 0 μM

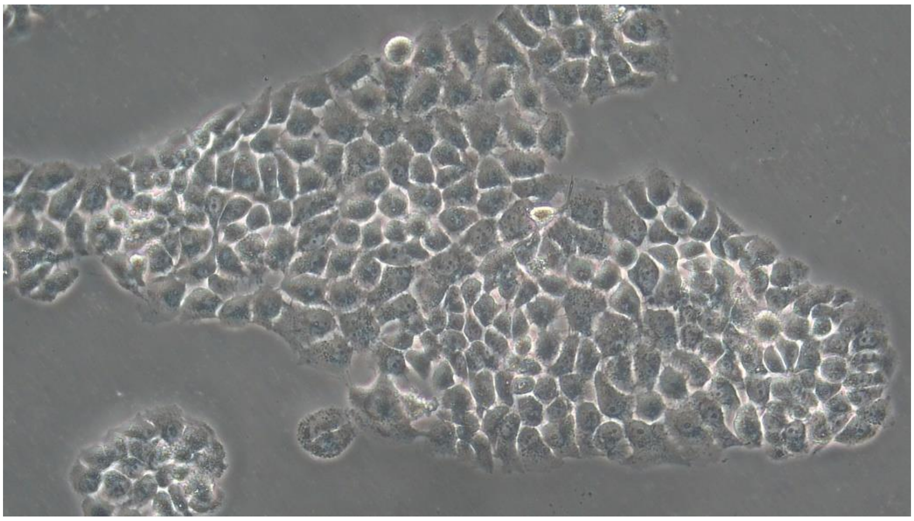

(b) 25 μM

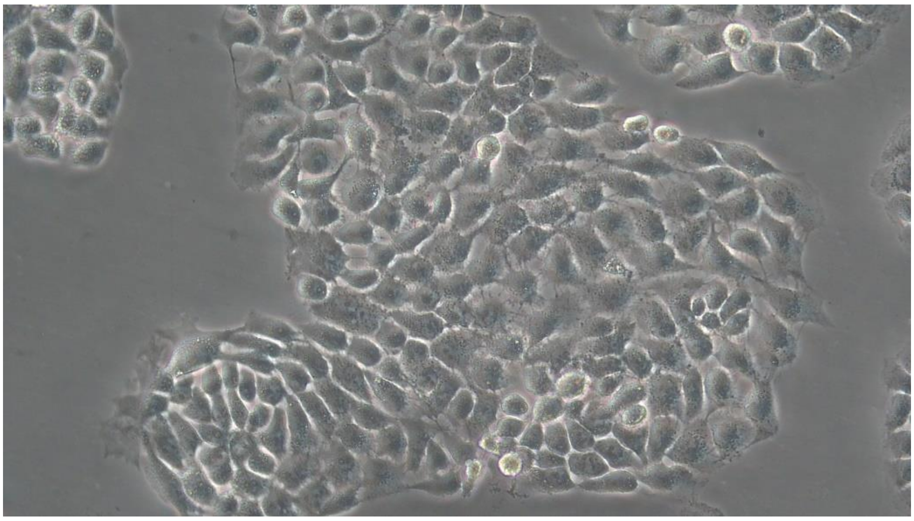

(c) 50 μM

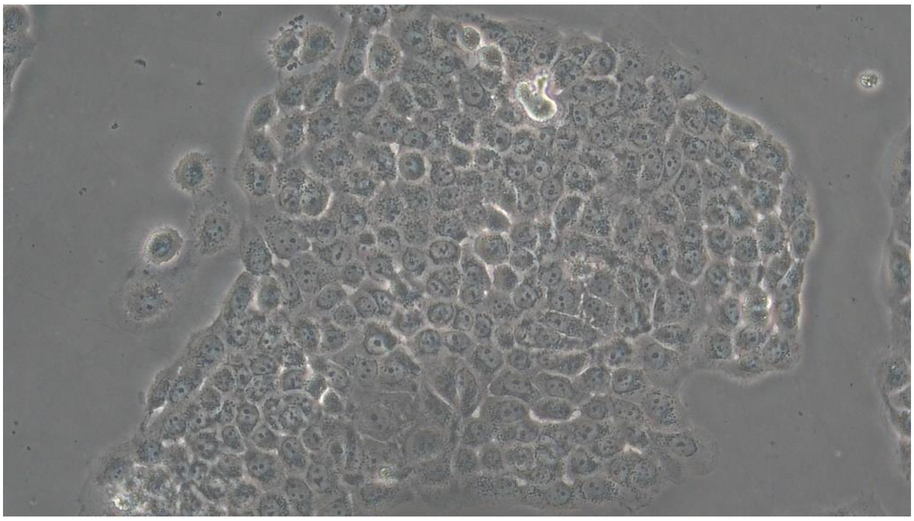

(d) 100 μM

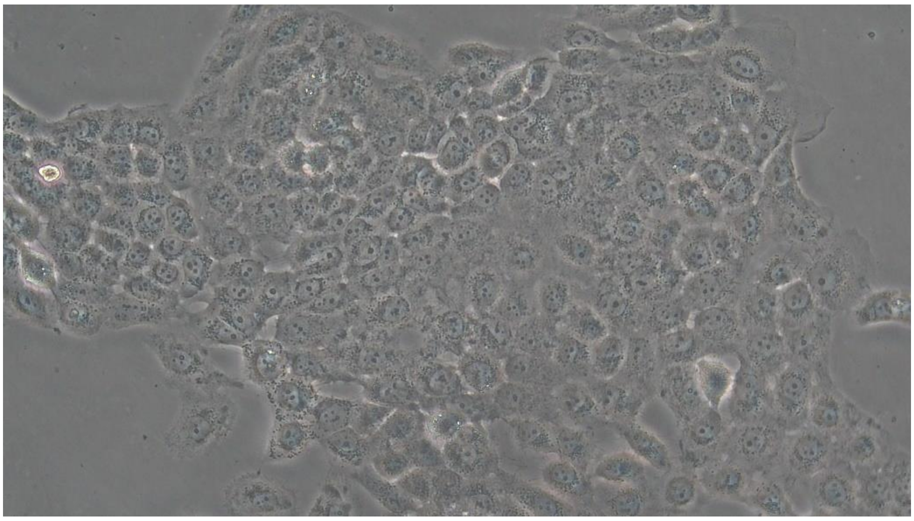

(e) 0 μM

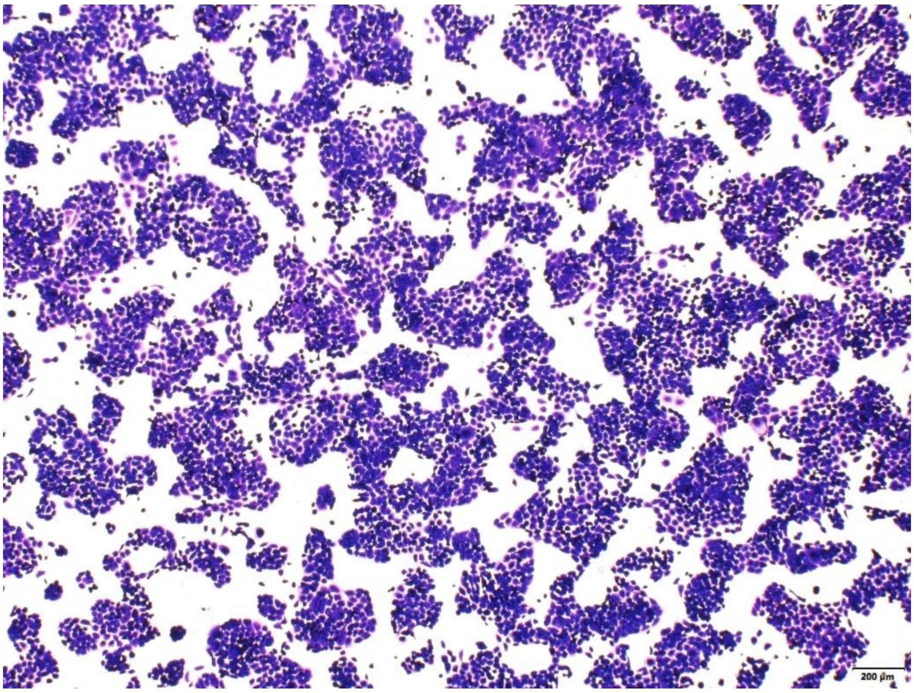

(f) 25 μM

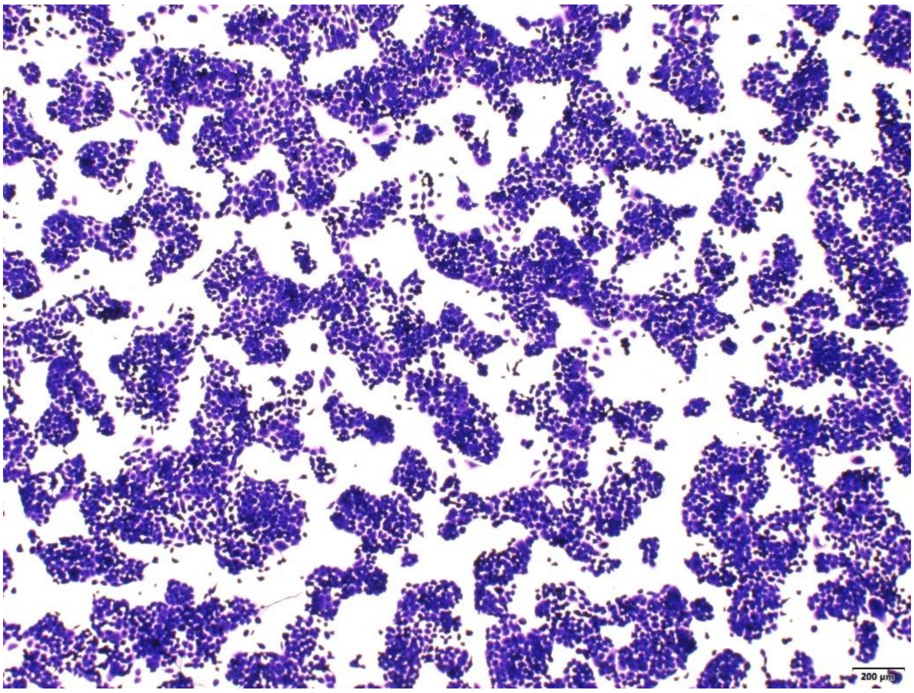

(g) 50 μM

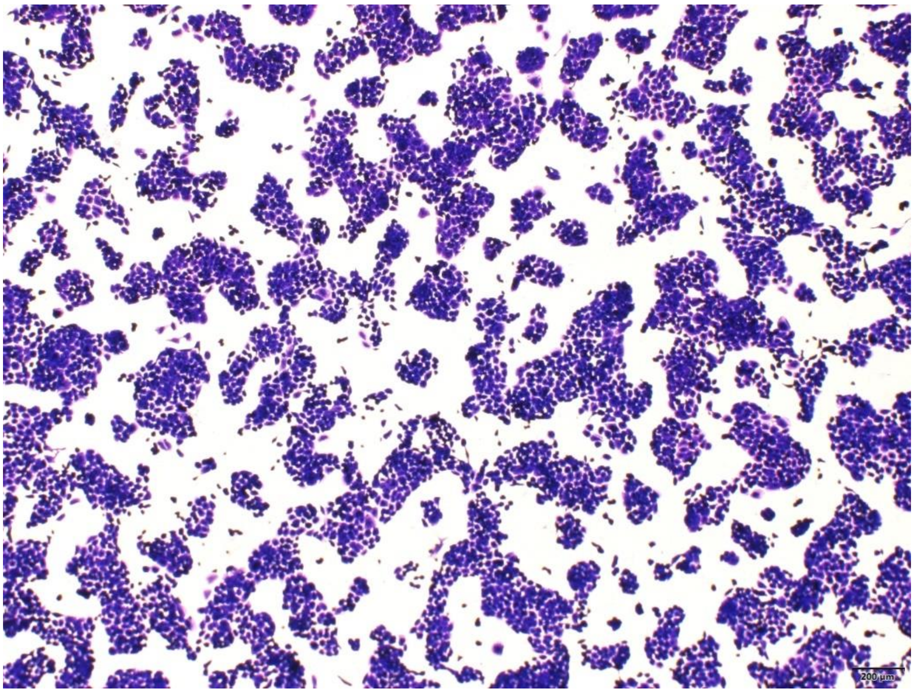

(h) 100 μM

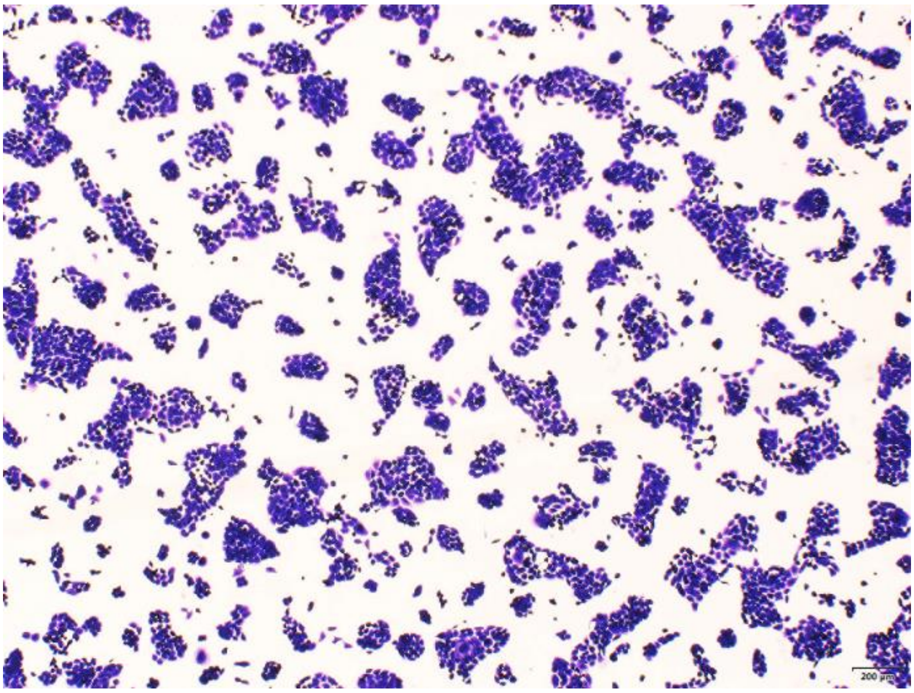

Supplementary Figure S8. The raw microscopic images for Figure 1c. (a-d) Observation of cellular morphology of SZ95 sebocytes exposed to various doses of emodin. original magnification, ×200. (e-h) Crystal violet staining for colony formation detection. Scale bar = 200 μm.

(a) 0 µM

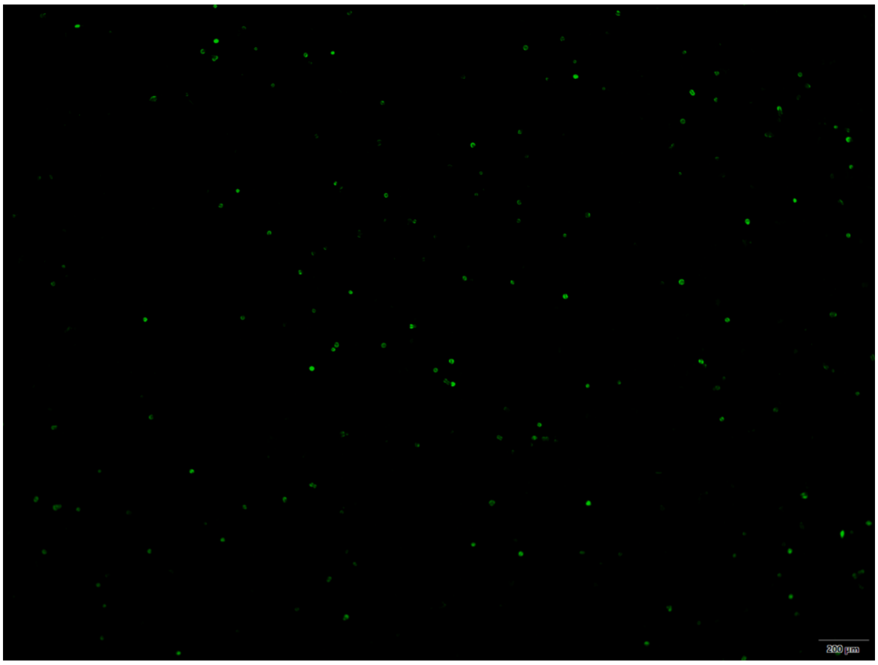

(b) 25 µM

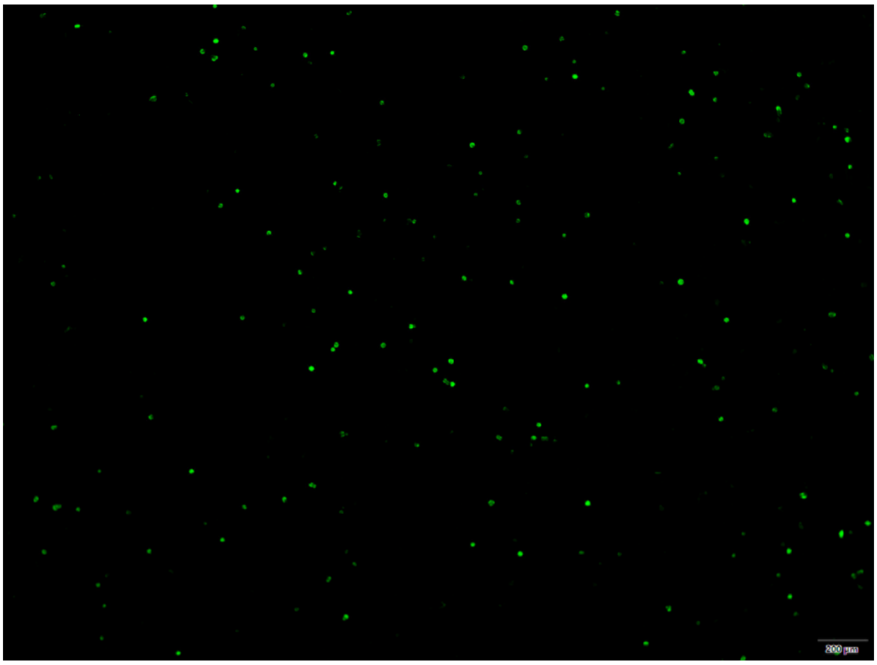

(c) 50 µM

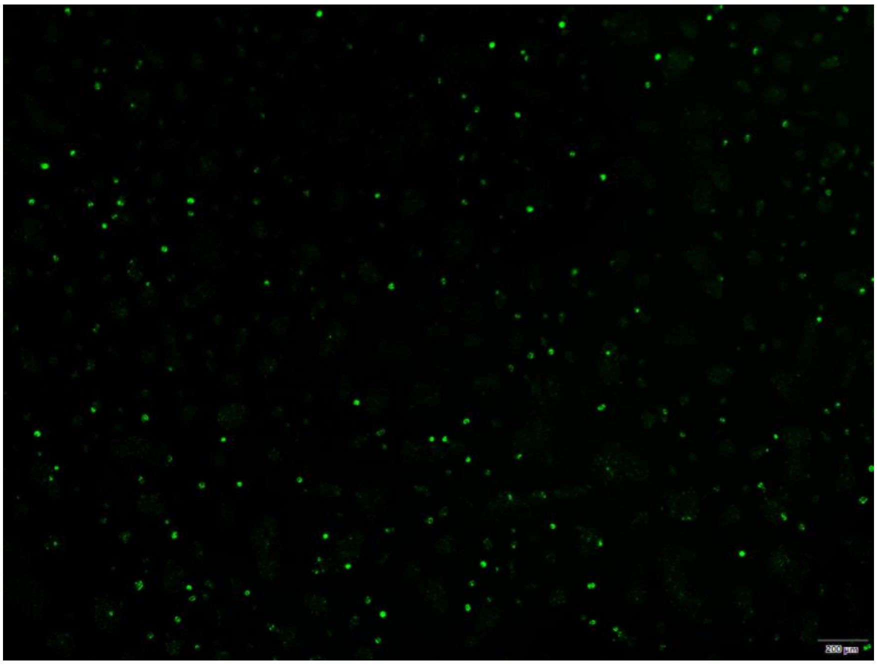

(d) 100 µM

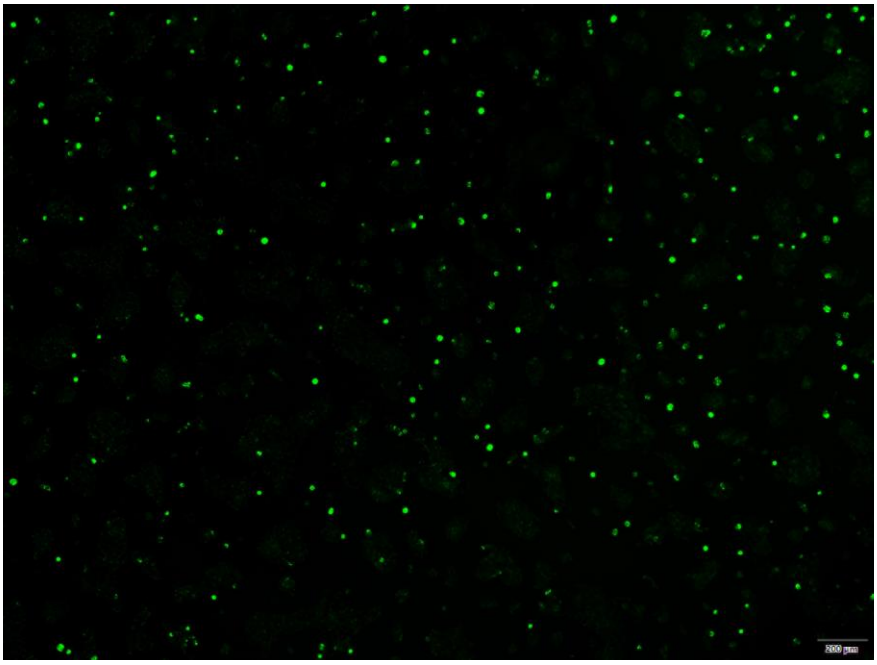

Supplementary Figure S9. The raw microscopic images for Figure 2b. Scale bar = 200 µm.

(a) 0 µM

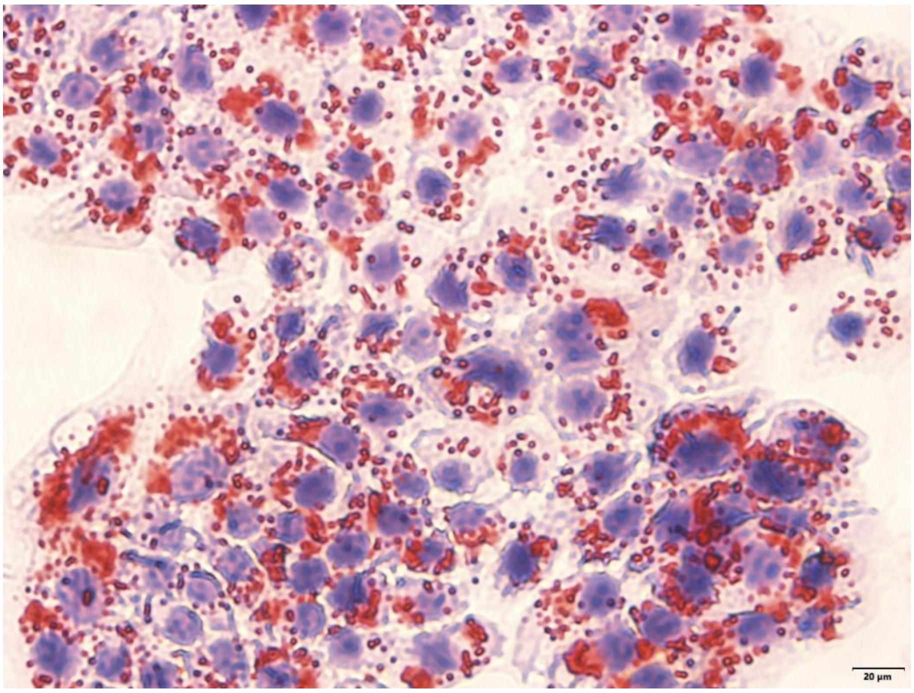

(b) 25 µM

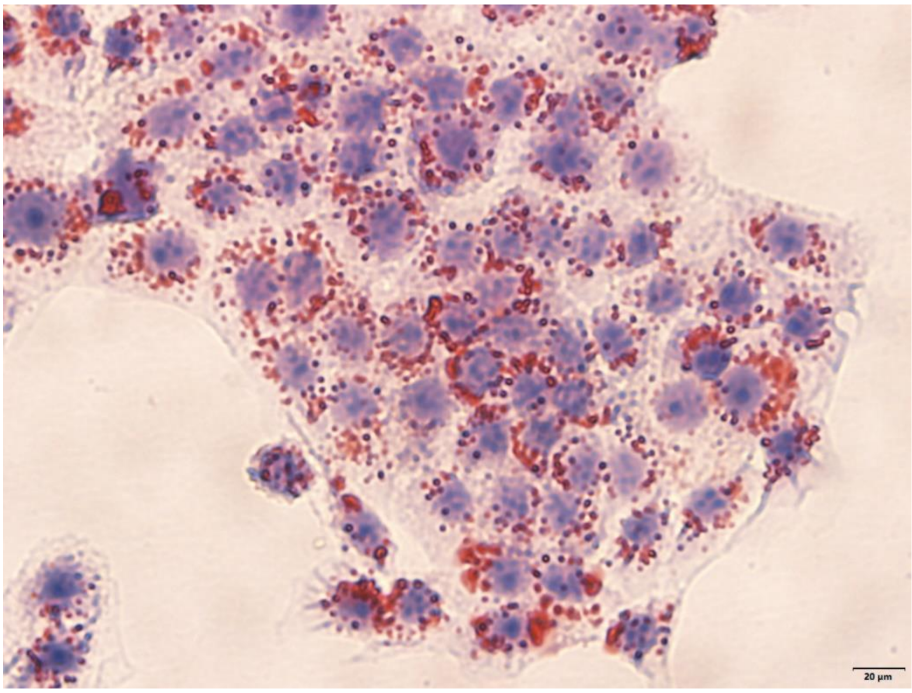

(c) 50 μM

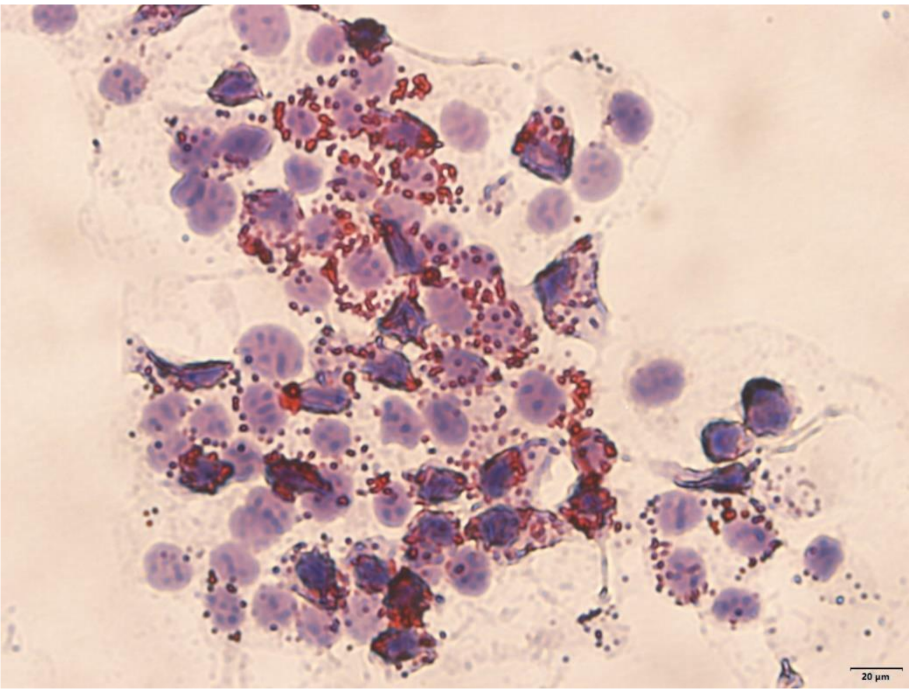

(d) 100 μM

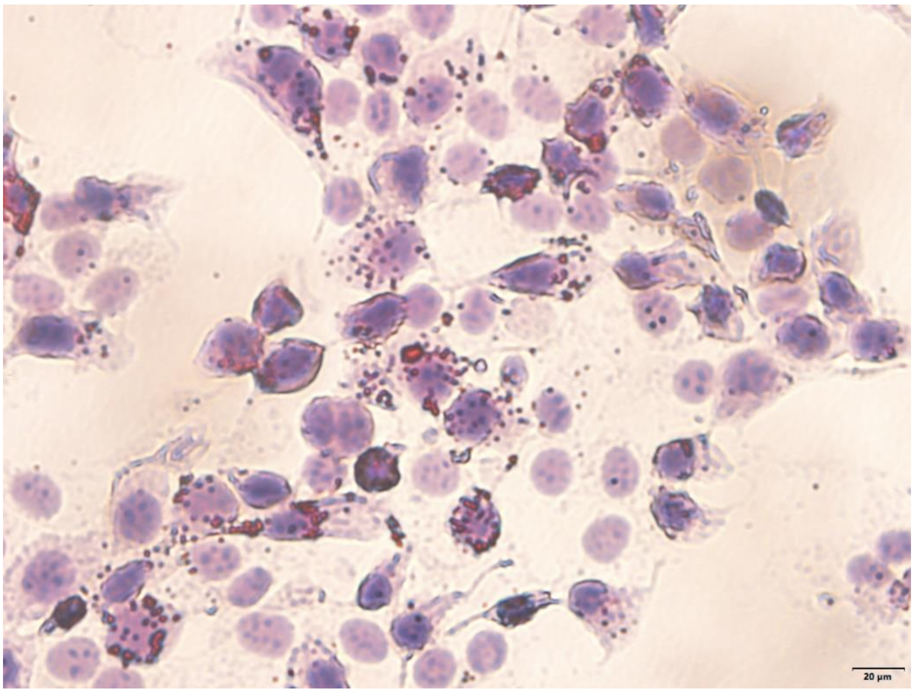

(e) Control

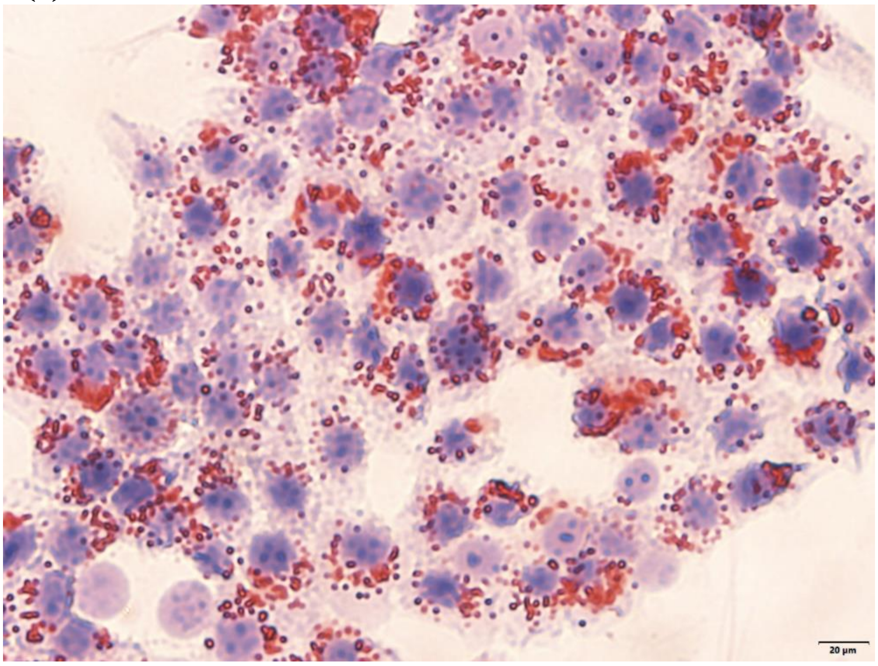

(f) IGF-1

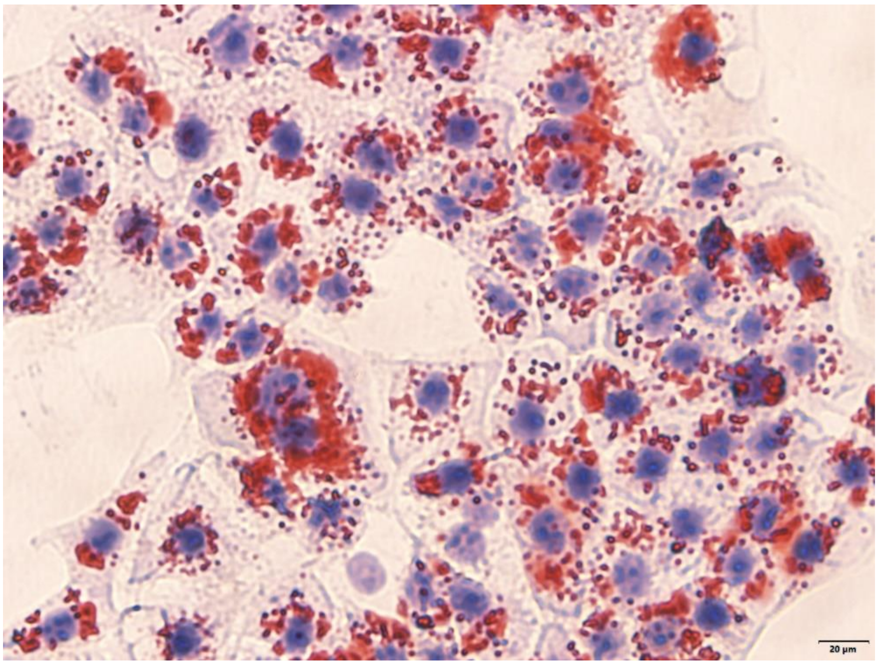

(g) IGF-1+emodin

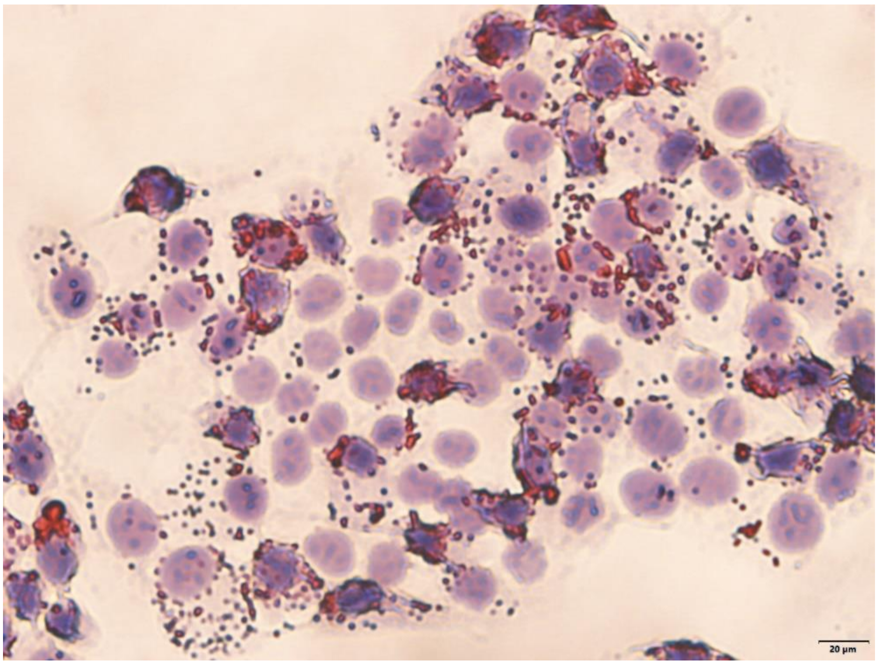

(h) IGF-1+LY

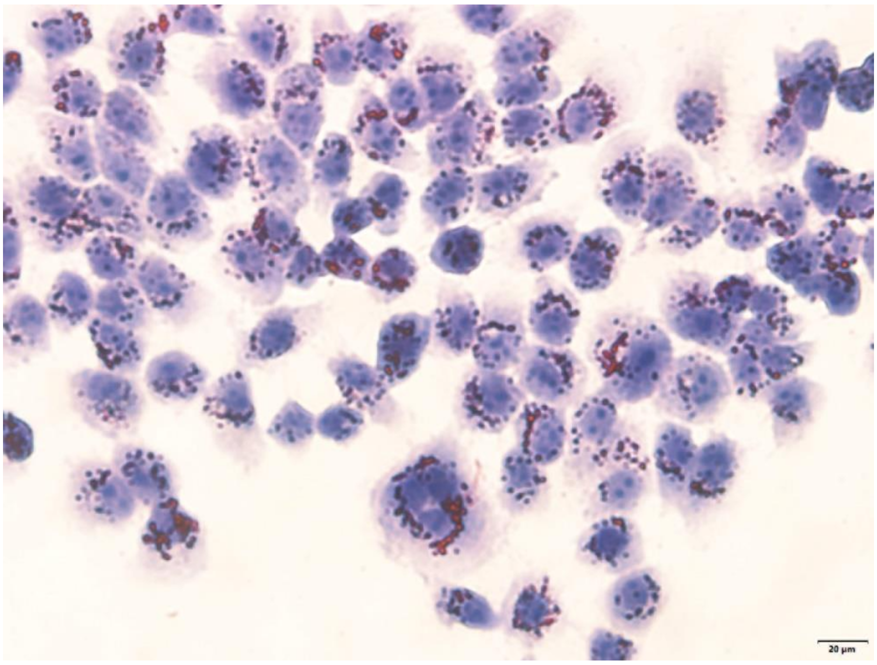

(i) IGF-1+LY+Emodin

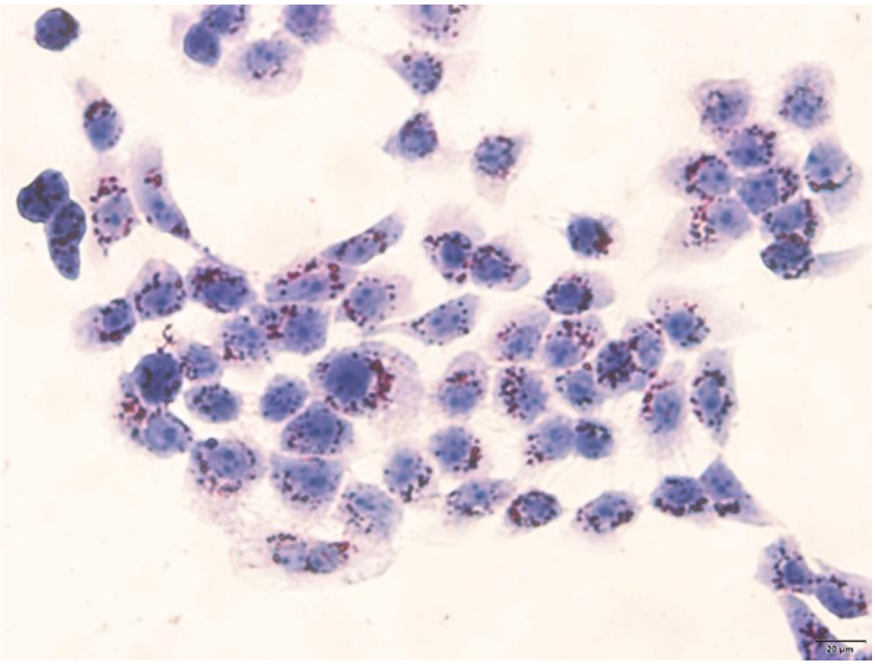

(j) IGF-1+13-*cis* RA

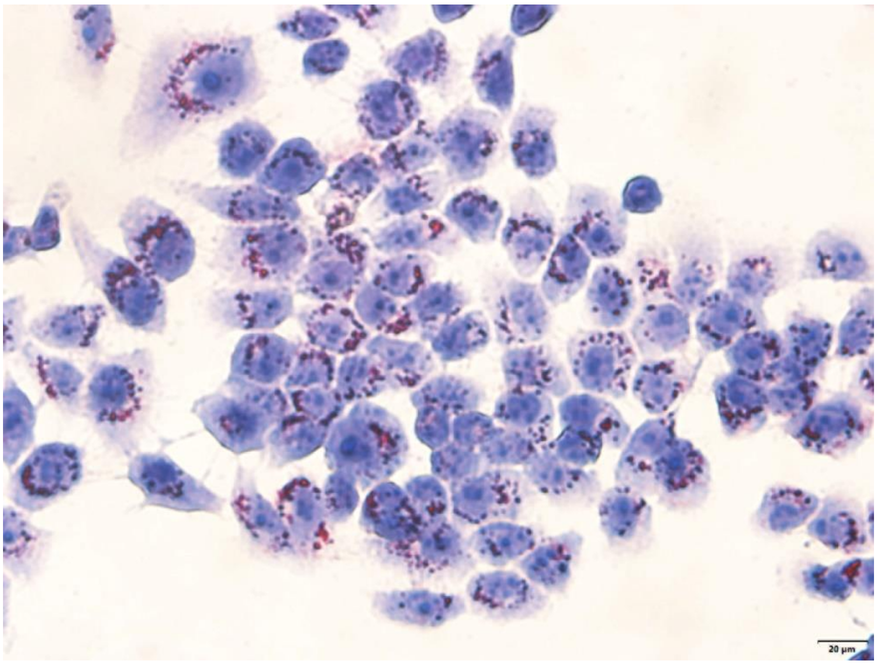

(k) IGF-1+13-*cis* RA+Emodin

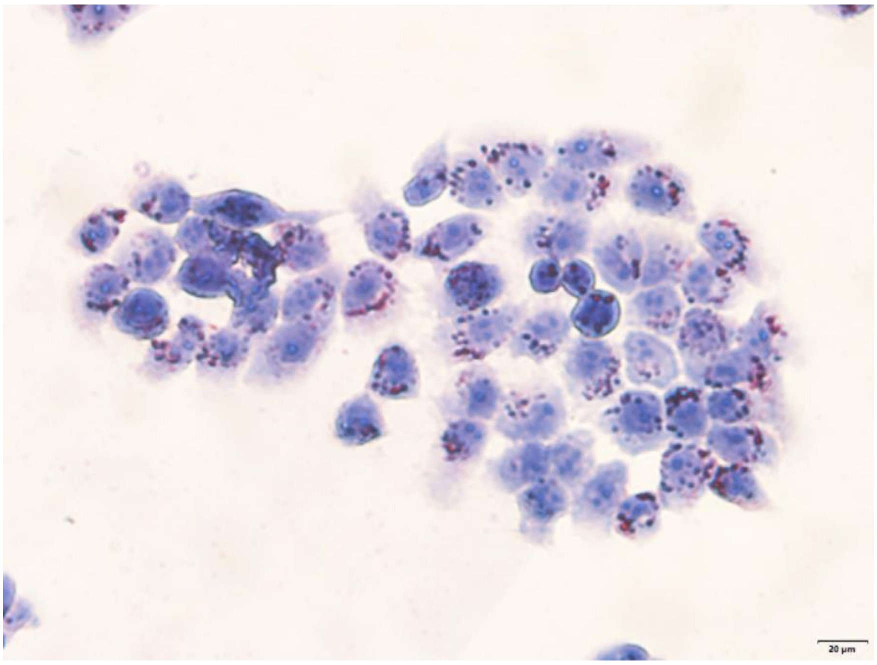

Supplementary Figure S10. The raw data for microscopy of Oil Red O staining. (a-d) The original microscopic images for Figure 3a. (e-k) The original microscopic images for Figure 3d.
